# Host SNARE Proteins Mediate Lysosome and PVM Fusion to Support *Plasmodium* Liver Infection

**DOI:** 10.3390/cells15070584

**Published:** 2026-03-25

**Authors:** Kodzo Atchou, Nicolas Kramer, Annina Bindschedler, Jacqueline Schmuckli-Maurer, Reto Caldelari, Volker T. Heussler

**Affiliations:** 1 Institute of Cell Biology, University of Bern, Baltzerstrasse 4, 3012 Bern, Switzerland; jacqueline.schmuckli@unibe.ch (J.S.-M.); reto.caldelari@unibe.ch (R.C.); 2 Graduate School for Cellular and Biomedical Sciences, University of Bern, Mittelstrasse 43, 3012 Bern, Switzerland; nicolas.kramer@unibas.ch (N.K.); bindschedler.a@gmail.com (A.B.)

**Keywords:** *Plasmodium*, liver infection, host SNARE, lysosomes, PVM, membrane fusion, CRISPR/Cas9

## Abstract

**Highlights:**

**What are the main findings?**

- Host endolysosomal SNARE proteins are sequentially recruited to the *Plasmodium* parasitophorous vacuole membrane during liver-stage infection, with early incorporation of VAMP7 and Vti1B followed by later recruitment of VAMP8 and Stx7.
- Combinatorial SNARE knockouts reveal functional redundancy but identify Vti1B-containing complexes as critical for lysosome–PVM fusion, parasite growth, and survival.

**What are the implications of the main findings?**

- These findings demonstrate that *Plasmodium* hijacks host SNARE-mediated lysosomal trafficking to acquire host-derived resources essential for liver-stage development.
- Targeting host SNARE complexes, particularly those containing Vti1B, offers a promising host-directed strategy to impair parasite development while limiting the potential for drug resistance.

**Abstract:**

Malaria, caused by *Plasmodium* parasites, remains a global health crisis, necessitating novel therapeutic strategies targeting host–parasite interactions. During liver-stage infection, parasites exploit host vesicular trafficking machinery, particularly SNARE (soluble N-ethylmaleimide-sensitive factor attachment protein receptor) proteins that mediate membrane fusion. Using a CRISPR/Cas9 knockout system in HeLa cells combined with advanced microscopy of *Plasmodium berghei*-infected HeLa cells, we identified specific endolysosomal SNAREs including Vesicle-Associated Membrane Protein 7 (VAMP7), Vesicle-Associated Membrane Protein 8 (VAMP8), Vesicle Transport Through Interaction With T-SNAREs 1B (Vti1B), and Syntaxin 7 (Stx7) to be recruited to the parasitophorous vacuole membrane (PVM) with distinct temporal profiles. This demonstrates the parasite’s precise manipulation of host endolysosomal trafficking pathways. VAMP7 and Vti1B were localized to the PVM within 30 min post-infection, suggesting potential roles during invasion, while VAMP8 and Stx7 appeared later around 24 h post infection (hpi), coinciding with increased nutrient acquisition. Single gene deletions showed minimal impact, but combinatorial knockouts (KO) revealed critical redundancy. VAMP7-VAMP8 as well as VAMP7–Vti1B double KO significantly reduced parasite infection and growth, with Vti1B playing a dominant role. Triple KO phenotypes mirrored VAMP7-Vti1B disruption, underscoring Vti1B’s dominant role. SNARE depletion also impaired the lysosome–PVM association and LAMP1 positive vesicle recruitment. Our findings indicate *Plasmodium* hijacks a coordinated host SNARE network to fuse lysosomes with the PVM for nutrient uptake. Targeting Vti1B-containing complexes disrupts this pathway without host cell toxicity, offering a promising host-directed antimalarial approach.

## 1. Introduction

Malaria remains one of the most devastating global health challenges, with an estimated 263 million cases and 597,000 deaths reported in 2023, the majority occurring in sub-Saharan Africa [1]. The disease is caused by *Plasmodium* parasites transmitted through infected *Anopheles mosquitoes*. Following transmission, sporozoites travel to the liver where they invade hepatocytes and undergo extensive replication, producing thousands of merozoites that subsequently initiate the symptomatic blood-stage infection [2,3,4]. Among the five *Plasmodium* species that infect humans, *Plasmodium falciparum* (*P. falciparum*) is the most virulent, and the emergence of drug resistance particularly to artemisinin-based therapies continues to undermine control efforts [5]. These challenges highlight the urgent need for novel intervention strategies targeting understudied aspects of the parasite life cycle, particularly the clinically silent liver stage.

During liver-stage infection, only a small number of hepatocytes become infected. Within these cells, the parasite undergoes extensive asexual schizogony, producing thousands of new daughter merozoites [3,4]. While this replication process is remarkably efficient, the parasite relies entirely on host cell resources for its growth and development. During its development within hepatocytes, the parasite resides within a parasitophorous vacuole (PV) enclosed by the PVM. This specialized compartment is crucial for nutrient acquisition, immune evasion, and parasite egress [6,7,8,9]. The host hepatocyte serves as a protective niche, shielding the parasite from immune detection while supplying essential nutrients. For instance, *Plasmodium* lacks the ability to synthesize sterols *de novo* and instead scavenges host-derived cholesterol, either produced internally or acquired via low-density lipoproteins [10]. Additionally, the parasite relies on soluble host factors, such as proteins and solutes, to support its replication during schizogony. These nutrients may be delivered via the host cell’s canonical autophagy and endolysosomal pathways. Various host vesicles including autophagosomes, amphisomes, late endosomes, and lysosomes have been observed in associated with or near the PVM, and disrupting these compartments impairs robust liver-stage development [8,11,12,13,14,15]. Despite the well-established importance of the PVM, the molecular mechanisms underlying its interactions with host organelles, particularly lysosomes and late endosomes, remain poorly understood. Intriguingly, many intracellular pathogens manipulate host vesicle trafficking to promote their survival and replication [16,17,18]. This raises the possibility that *Plasmodium* similarly exploits host membrane trafficking machinery, particularly SNARE-mediated fusion events, to facilitate its growth within hepatocytes.

SNARE proteins are a highly conserved family of membrane-associated proteins that mediate vesicle fusion in eukaryotic cells, playing essential roles in endocytosis, exocytosis, autophagy, and lysosomal delivery [19,20]. They are broadly classified into vesicle-associated SNAREs (v-SNAREs) and target membrane-associated SNAREs (t-SNAREs) [21,22]. v-SNAREs typically contribute an arginine (R) residue to the fusion process, whereas the t-SNAREs typically contribute a glutamine (Q) residue. During fusion, a v-SNARE on the vesicle forms a stable four-helix bundle with three t-SNAREs on the target membrane, driving membrane proximity and overcoming the energy barrier to fusion [23]. SNAREs are widely distributed across eukaryotes, with 38 members in humans, 25 in *Saccharomyces cerevisiae*, and 18 SNARE-like proteins in *Plasmodium* [24,25]. In mammalian cells, specific SNAREs including VAMP7, VAMP8, and Vti1B regulate endolysosomal trafficking. These proteins often function in precise combinations to ensure the specificity of membrane fusion and cargo delivery [26,27]. SNARE proteins, as key regulators of vesicle trafficking, are frequently exploited by intracellular pathogens. Many parasites, bacteria and viruses have developed strategies to manipulate SNARE function, diverting vesicular transport to facilitate their replication or avoid immune detection [16,28,29,30,31,32]. However, the role of host SNARE proteins in *Plasmodium* liver-stage development remains poorly understood. Notably, none of the SNARE-like proteins investigated so far in *Plasmodium* localize to the PVM, suggesting that the parasite may instead co-opt host SNARE machinery to mediate the fusion of nutrient-carrying vesicles. Investigating how *Plasmodium* engages these host factors could yield crucial insights into the molecular mechanisms driving its intracellular survival and a novel target for antimalarial intervention.

Using a CRISPR/Cas9-engineered knockout system combined with quantitative imaging, this study aims to identify the key SNARE proteins that mediate vesicle fusion with the PVM during *Plasmodium* liver-stage development, focusing on those involved in late endosomal and autophagic pathways.

## 2. Materials and Methods

### 2.1. Ethics Statement

The experiments were conducted in strict accordance with the Swiss Animal Protection Act (TSchG) and received approval from the Animal Experimentation Commission of the Canton of Bern (Authorization BE118/22). Research staff involved in animal handling possessed certifications accredited by the Federation of European Laboratory Animal Science Associations (FELASA). Female C57BL/6 and BALB/c mice, aged 6 to 12 weeks and weighing 20–30 g at the time of infection, were obtained from Janvier Labs (France) or bred in-house at the University of Bern’s Institute of Cell Biology (IZB). Housing was provided in individually ventilated cages (IVCs) containing standard bedding and nesting material. Environmental conditions were maintained at a 12 h light/dark cycle, 20–22 °C, and 55 ± 10% relative humidity. Ad libitum access to chow and water was provided, and animal well-being was ensured through daily monitoring by trained personnel.

### 2.2. Plasmodium Parasite Lines

The study used the *P. berghei* ANKA strain, including fluorescent reporter lines *Pb*mCherry (expressing mCherry under *P. berghei* hsp70) [9].

### 2.3. Cell Culture and In Vitro Infection of HeLa Cells

Human epithelial HeLa cells (European Cell Culture Collection) were cultured in complete Minimum Essential Medium (cMEM; MEM with Earle’s salts (1–31F01-I, BioConcept, Allschwil, Switzerland) supplemented with 10% heat-inactivated fetal bovine serum [Sigma-Aldrich, Buchs, Switzerland], 100 U penicillin, 100 µg/mL streptomycin and 2 mM L-Glutamine (all from BioConcept, Allschwil, Switzerland) at 37 °C under 5% CO_2_ atmosphere. For infection assays, 40,000 HeLa cells were seeded onto glass coverslips in 24-well plates, allowed to adhere overnight, then infected with *P. berghei* sporozoites in cMEM containing 2.5 μg/mL amphotericin B (E437, Bioconcept, Allschwil, Switzerland) to prevent fungal contamination, with medium changes performed every 24 h.

### 2.4. Stable Expression of GFP-Tagged SNARE Constructs in HeLa Cells

To generate HeLa cells stably expressing GFP-tagged SNAREs, SNARE-GFP constructs were subcloned into lentiviral backbone plasmids (pLX307, Addgene #98343 or pLVX, Addgene #58263, Watertown, MA, USA) as previously described [33]. Briefly, the open reading frames of VAMP7, VAMP8, and Stx7 were amplified using specific primers (VAMP7-GFP: 5′-GATCCGCTAGCGCTACCGG-3′ and 5′-CTATTTCTTCACACAGCTTGGCCATG-3′; VAMP8-GFP: 5′-GATCCGCTAGCGCTACCGG-3′ and 5′-TTACTTGTACAGCTCGTCCATGCC-3′; Stx7-GFP: 5′-ATGTTTACACTCCAGGAGTTGG-3′ and 5′-AGATATCTTTACTTGTACAGCTCGTCCATG-3′) and cloned via NheI and EcoRV restriction sites. The lentiviral plasmid pLX307 (Addgene #98343, Watertown, MA, USA) was kindly provided by William Hahn and Sefi Rosenbluh [34,35], while the VSV-G envelope plasmid pMD2.G (Addgene #12259, Watertown, MA, USA) and packaging plasmid psPAX2 (Addgene #12260, Watertown, MA, USA) were gifts from Didier Trono. Lentiviruses (Addgene #58263, Watertown, MA, USA) were produced in human embryonic kidney (HEK293T) cells, and HeLa cells were transduced following established protocols [33]. Transduced cells were selected with 1 µg/mL puromycin from 2 days post-transduction for 3 days, and single-cell clones were isolated and validated by live imaging and immunofluorescence assay (IFA) using anti-GFP antibodies.

### 2.5. Generation of SNARE Knockout Cell Lines Using CRISPR/Cas9

SNARE knockout (KO) cell lines were generated using the CRISPR/Cas9 GeCKO v2 lentiviral system [36]. The system employed two vectors: pLentiCas9-Blast (blasticidin resistance; encoding hSpCas9; Addgene #52962, Watertown, MA, USA) and pLentiGuide-Puro (puromycin resistance; encoding sgRNAs; Addgene #52963, Watertown, MA, USA), both gifts from Feng Zhang. Target-specific guide RNAs were designed to disrupt exon 1 of human VAMP7 (gRNA: caccgAACAGCAAAAAGAATCGCCA), exon 2 of VAMP8 (gRNA: caccgTGTGCGGAACCTGCAAAGTG), exon 1 of Vti1B (gRNA: caccgAAGCTGCACGAGATCTTCCG), and exon 3 of Stx7 (gRNA: caccgGATGTTAGAAGAGATCCTCT), and were cloned into the pLentiGuide-Puro backbone. For double (2KO) or triple (3KO) knockouts, we additionally used lentiGuide-Hygro-eGFP (hygromycin resistance; Addgene #99375, Watertown, MA, USA) or lentiGuide-zeo (zeocin resistance; Addgene #99375, Watertown, MA, USA). Lentiviruses were produced as previously described [33], and HeLa cells were transduced with both Cas9- and sgRNA-encoding viruses. Transduced cells were selected with puromycin (1 μg/mL) and blasticidin (4 μg/mL) for 3 days, or with hygromycin/zeocin (500 μg/mL) for 5 days when using hygro-eGFP or zeocin-based constructs. For combinations of resistance markers (Puro/Hygro/Zeo/Blast), antibiotics were applied simultaneously with daily media changes and cell death monitoring. Single-cell clones were isolated and validated by western blotting and Sanger sequencing of target loci using gene-specific primers (VAMP7: 5′-CTTCTGTTGAACTATTTCTCTAGG-3′ and 5′-ACCAGATATATTTTCTAAGTTGGTG-3′; VAMP8: 5′-CATCTCACCTTCTGGGGCTTACAAATGC-3′ and 5′-GTCATGGGATCCATTCCCTGGGATCC-3′; Vti1B: 5′-CTTTCGCTGCGGCCTTTCCCCAAC-3′ and 5′-GGACCGCAGATGCAAAAGCCCCTC-3′; Stx7: 5′-GACTCTGTCCTTTGAAGTCCAG-3′ and 5′-GTAGTGCTACCTGGTTCCAAAC-3′). Editing efficiency was quantified using TIDE (https://tide.nki.nl, accessed on 13 June 2022) where applicable.

### 2.6. Western Blotting

Confluent HeLa cells in 6-well plates were rinsed with PBS and lysed in 200 μL of preheated (95 °C) Laemmli buffer (25 mM Tris-HCl pH 6.8, 2% glycerol, 0.8% SDS, 0.004% bromophenol blue, 2% 2-mercaptoethanol). Lysates were collected, treated with Universal Nuclease (Thermo Fisher #88701, Reinach, Switzerland; 5 min, RT), and heat-denatured at 95 °C for 5 min as previously described [35]. Proteins were resolved on 15% SDS-PAGE gels alongside PageRuler Pre-stained NIR markers (Thermo Fisher #26635, Reinach, Switzerland) and transferred to nitrocellulose membranes using a Hoefer tank blot system. After blocking for 30 min with 5% non-fat milk in PBST (PBS containing 0.05% Tween-20), membranes were incubated for 1 h at room temperature with primary antibodies: mouse anti-VAMP7 (1:500; Synaptic Systems #232011, Goettingen, Germany), mouse anti-VAMP8 (1:500; Santa Cruz #sc-166820, Heidelberg, Germany), mouse anti-Vti1B (1:1000; BD Biosciences #611404, Allschwil, Switzerland), mouse anti-Stx7 (1:500; Santa Cruz #sc-514017, Heidelberg, Germany), chicken anti-GAPDH (1:3000; Millipore #AB2302, Germany) and mouse anti-GAPDH (1:3000; DSHB #2G7, Iowa City, IA, USA), and mouse anti-α-tubulin (1:1000; Cell Signaling #2125, Allschwill, Switzerland). Membranes were incubated for 1 h with either HRP-conjugated secondary antibodies goat anti-mouse IgG (HRP) (1:3000; Sigma-Aldrich #A5278, Buchs, Switzerland), and goat anti-chicken IgY (HRP) (1:5000; Invitrogen #A16054, Reinach, Switzerland) or with fluorescent secondary antibodies: green (goat anti-mouse, 1:5000; LI-COR #926-32210, Bad Homburg, Germany) and red (goat anti-mouse, 1:5000; LI-COR #926-68020, Bad Homburg, Germany). Protein signals were then detected using SuperSignal West Femto substrate (Thermo Fisher #34096, Reinach, Switzerland), and chemiluminescence was captured with an Amersham Imager 600 (Minneapolis, MN, USA) in semi-automated mode or with a LI-COR Odyssey imaging system (Lincoln, NE, USA).

### 2.7. Sporozoite Isolation from Mosquito Salivary Glands

Sporozoites were isolated from *Anopheles mosquitoes* using established protocols [37,38]. Mosquitoes were fed on *Plasmodium*-infected mice with 0.2–1% gametocytemia, and isolations were initiated from day 18 post-infection. For salivary gland sporozoite collection, mosquitoes were anesthetized with chloroform, surface-sterilized in 70% ethanol, and rinsed twice with PBS. Glands were dissected under a binocular microscope in Iscove’s Modified Dulbecco’s Medium (IMDM), pooled in 50 μL ice-cold IMDM, and homogenized using a motorized pestle system (Z359947/Z359971, Sigma, Buchs, Switzerland). The sporozoite suspension was quantified using a Neubauer hemocytometer by counting sporozoites in four corner grids. Total yields (sporozoites/mL and per mosquito) were calculated based on dilution factors and dissection counts. Freshly isolated sporozoites were immediately used to infect seeded HeLa cells and were centrifuged (1000× *g*, 1 min) before being incubated at 37 °C with 5% CO_2_.

### 2.8. Primary Hepatocyte Isolation

Primary mouse hepatocytes were isolated from 12- to 16-week-old Balb/c or C57BL/6 mice using a two-step perfusion method, as previously described [8,39]. Mice were euthanized by CO_2_ inhalation, and the liver was perfused via the portal vein or inferior vena cava, first with HEPES buffer (5 min) and subsequently with collagenase solution (7 min). Following perfusion, the liver was excised, mechanically dissociated in collagenase solution, and the resulting cell suspension was filtered and washed three times by centrifugation (50× *g*, 2 min) in William’s Medium E (WME). The final cell pellet was resuspended in complete WME medium (supplemented with L-glutamine, penicillin–streptomycin, amphotericin B, insulin–transferrin, and aprotinin). Cells were counted and seeded at appropriate densities. After 1 h of adhesion, the culture medium was replaced or supplemented after 2–4 h.

### 2.9. Live Cell Imaging

HeLa cells were seeded in 96-well plates and allowed to adhere overnight before infection with *P. berghei* salivary gland sporozoites during a 2 h incubation at 37 °C. Following infection, cells were washed twice with MEM medium to remove uninternalized sporozoites and salivary gland debris, and the cells were then rinsed with 1XPBS and incubated with accutase for 10 min before being transferred to 24-well glass-bottom imaging plates (P24-1.5H-N, CellVis, Mountain View, CA, USA) for maintenance in complete cMEM at 37 °C/5% CO_2_ as previously described [8,40]. After 1 h incubation in fresh cMEM, time-lapse imaging was performed using a Nikon W1 LIPSI spinning-disk confocal system (Amstelveen, The Netherlands) with a 100 oil-immersion objective (NA 1.45), capturing infected cells at defined intervals to simultaneously monitor the infected cells dynamics and parasite-tagged protein fluorescence signal development throughout the intracellular lifecycle.

### 2.10. Immunofluorescence Analysis

Immunofluorescence was carried out as previously described [41,42]. Infected HeLa cells on glass coverslips were fixed with 4% PFA (F8775, Sigma, Buchs, Switzerland) in PBS at room temperature for 15 min, washed with PBS, and blocked with 10% FCS/PBS for 20 min at room temperature. The cells were then incubated in 10% FCS/PBS for 1 h, at room temperature with primary antibodies: Vti1B (mouse mAb 1:1000 (1:500 PS-ExM), BD Transduction Laboratories #611404, Allschwil, Switzerland), UIS4 (rabbit 1:1000, *P. sinnis*; chicken 1:10000, Proteogenix, Schiltigheim, France), hLAMP1 (mouse mAb 1:1000 (1:500 PS-ExM), DSHB H4A3 (Iowa City, USA); rabbit pAb 1:1000, Cell Signaling #9091, Allschwill, Switzerland), GFP (rabbit pAb 1:1000, Origene TA100030, Herford, Germany; mouse mAb 1:1000, Roche AQ160, Basel, Switzerland), and α-tubulin (guinea pig pAb 1:500, ABCD AA345, Geneva, Switzerland). After PBS washes, the cells were incubated in secondary antibodies (1:1000, 1 h room temperature) including: anti-rabbit/mouse Alexa Fluor 488/594 (Invitrogen A11034/A21207/A11001/A11032, Reinach, Switzerland), anti-guinea pig Alexa Fluor 647 (Sigma-Aldrich SAB4600033, Buchs, Switzerland), and anti-chicken Cy5 (Jackson ImmunoResearch #AB_2340365). Nuclei were stained with DAPI before mounting with ProLong Gold (Invitrogen #P36930, Reinach, Switzerland). The cells were imaged with a Nikon W1 LIPSI Confocal microscope with DAPI/FITC/TRITC/Cy5 filters (Amstelveen, The Netherlands). The single-staining controls were conducted to exclude crosstalk.

### 2.11. Pre-Staining Expansion Microscopy (PS-ExM)

PS-ExM of infected HeLa cells expressing SNARE proteins was performed as previously described [43]. Briefly, cells grown on 13 mm glass coverslips in 24-well plates were fixed with 4% PFA in PBS. Pre-expansion immunofluorescence staining was carried out as described above. The cells were then anchored in PBS containing 0.7% formaldehyde and 1% acrylamide at 37 °C for 2 h. Gelation was carried out using a monomer solution (MS) containing 19% sodium acrylate (408220, Sigma, Buchs, Switzerland), 10% acrylamide (A4058, Sigma, Buchs, Switzerland), and 0.1% bisacrylamide (M1533, Sigma, Buchs, Switzerland). The MS was mixed with 10% TEMED (17919, Thermo Fisher, Reinach, Switzerland) and 10% APS (5 µL each per 90 µL monomer solution). Samples on coverslips were inverted onto 35 µL monomer droplets on parafilm in a humid chamber, incubated on ice for 5 min to improve penetration, and polymerized at 37 °C for 30 min. Gel-embedded samples were denatured in SDS buffer (200 mM SDS, 200 mM NaCl, 50 mM Tris-HCl, pH 9) with agitation (200 rpm, 15 min at room temperature), followed by incubation at 95 °C for 1 h. Initial expansion was performed in MilliQ water for 1 h, with optional water exchange. The gels were incubated with DAPI (1 µg/mL in 2% BSA) to stain the DNA at 37 °C for 2 h. Fully expanded samples were mounted in 0.2% propyl gallate (02370, Sigma, Buchs, Switzerland) on poly-D-lysine-coated glass-bottom dishes (Gibco A38904, Buchs, Switzerland) and imaged using a Nikon W1 LIPSI confocal microscope (Amstelveen, The Netherlands).

### 2.12. Liver-Stage Parasite Size Measurement

To assess liver-stage parasite growth dynamics, HeLa cells were seeded at 40,000 cells/well (triplicate) in 96-well plates and infected 24 h later with 15,000 *Pb*mCherry sporozoites/well. Following a 2 h infection period at 37 °C, cells were washed, accutase treated and redistributed across eight wells of two 96-well plates (4 wells/plate per replicate) to achieve optimal imaging density [4,44]. Parasite development was monitored at 6, 24, and 48 hpi using an InCell Analyzer 2000 high-content imaging system (10× objective). Image analysis parameters (kernel size, sensitivity, and size threshold) were optimized in InCell Developer software to accurately segment parasites from background. Growth metrics were derived from cross-sectional area measurements, while survival rates were normalized to 6 hpi parasite counts (set as 100% viability baseline).

### 2.13. Quantification of Lysosome (LE) and Late Endosome Association with Plasmodium PVM

To quantify lysosome (LE) and late endosome associations with the *Plasmodium* PVM, 3D rendering of UIS4 (PVM marker) and LAMP1 (lysosomal/LE marker) from confocal Z-stacks (10–15 µm; 20 parasites/condition; 3 replicates) was performed in infected HeLa cells using Imaris. Cells infected with *Pb*mCherry sporozoites were fixed with 4% PFA in PBS at 24 hpi, stained for UIS4, LAMP1, DAPI (DNA), and either α-tubulin or Alexa Fluor 488-phalloidin to delineate cell boundaries, then imaged with spinning disk confocal microscopy. UIS4 signals were reconstructed as isosurfaces (detail level 0.215; absolute intensity), while LAMP1-positive vesicles were detected as isospots (local contrast mode; non-expanded cells: XY = 0.4 µm, Z = 0.5 µm; expanded cells: XY = 1.5 µm, Z = 2.4 µm). PVM-proximal LAMP1-positive isospots (≤0 µm distance) were quantified using α-tubulin-defined cell borders to exclude neighboring uninfected cells. Data were exported with the attached LE proportion calculated as (LAMP1-positive isospots at PVM) divided by (total LAMP1-positive isospots per infected cell) [43].

### 2.14. Image Acquisition and Analysis

The live-cell imaging, expansion microscopy, and IFA data were acquired using a Nikon W1 LIPSI spinning disk confocal system, with images processed and analyzed in FIJI (Java 1.8.0_322 (64-bit), USA), Huygens Professional deconvolution software (v3.8; Scientific Volume Imaging, The Netherlands), or IMARIS 3D/4D analysis software (v10.2.0). Data were quantified and visualized using GraphPad Prism v10.4.1 (GraphPad Software, San Diego, CA, USA), with statistical significance determined by one-way ANOVA and appropriate post hoc tests (* *p* ≤ 0.05, ** *p* ≤ 0.01, *** *p* ≤ 0.001). All experiments in this study were performed in triplicate.

## 3. Results

### 3.1. SNARE Proteins Are Recruited to the Plasmodium PVM During Liver-Stage Infection

**Figure 1:**
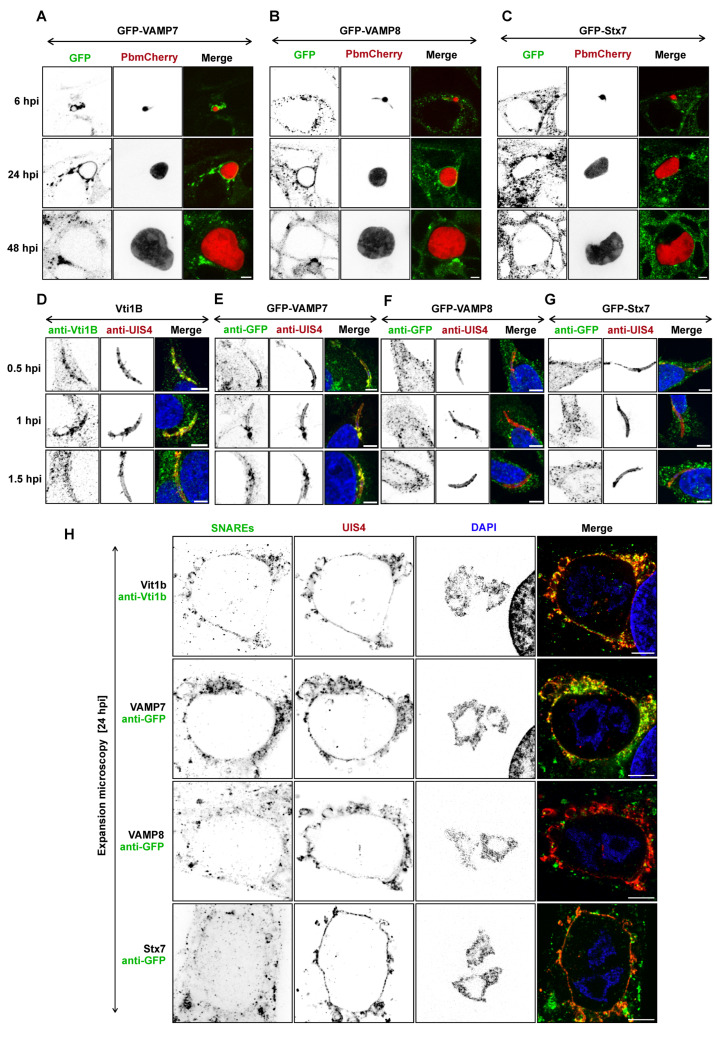
Localization of host SNARE proteins at the *Plasmodium* PVM during liver-stage infection. (**A**–**C**) Confocal live cell imaging of GFP-tagged SNARE proteins VAMP7-GFP (**A**), VAMP8-GFP (**B**), and Stx7-GFP (**C**) in *P. berghei*-infected HeLa cells expressing mCherry (parasite cytoplasm, red) at 6, 24, and 48 hpi. SNARE localization is shown in green. (**D**–**G**) Immunofluorescence analysis of Vti1B (**D**), VAMP7 (**E**), VAMP8 (**F**), and Stx7 (**G**) in HeLa cells fixed at 0.5, 1, and 1.5 hpi. SNAREs were detected with anti-GFP (green) or anti-Vti1B (green); the PVM was labeled with anti-UIS4 (red); and DAPI (blue) stained nuclei. (**H**) Localization of SNARE proteins (anti-Vti1B/anti-GFP in green) with the PVM marker UIS4 (red) at 24 hpi, visualized by expansion microscopy (5-fold expanded). Merged channels (yellow) highlight PVM-SNARE protein association. Nuclei were counterstained with DAPI (blue). Scale bars: 5 μm (**A**–**G**); 10 μm (**H**).

Previous studies have investigated the fusion of host late endosomal and lysosomal vesicles with the parasite PVM [11,12,15,43]. These fusion events were confirmed in the present study using expansion microscopy on infected HeLa cells fixed at 24 hpi (Supplementary Figure S1A,B). We next sought to determine the role of host SNARE proteins in mediating the fusion of host lysosomes with the parasite PVM during liver-stage infection. SNARE proteins involved in early-to-late endosomal and autophagosome–lysosome fusion were tagged and screened for PVM localization (Table 1). The association of SNARE proteins with the PVM was quantified using immunofluorescence and 3D Imaris software (v10.2.0), based on the proximity of the infected cell LAMP1-positive vesicles (defined as ≤0.5 µm from the PVM). From this analysis, four strongly (++ and +++) recruited candidates (VAMP7, VAMP8, Vti1B, and Stx7) were selected for in-depth investigation (Figure 1). Live cell imaging (Figure 1A–C) was performed on HeLa cells expressing GFP-tagged SNARE proteins. An exception was Vti1B, for which GFP tagging impaired proper protein folding. Endogenous Vti1b was therefore detected using an anti-Vti1b antibody or by expressing a Flag-tagged construct (Table 1). VAMP7 (Figure 1A) was strongly recruited to the parasite at 6 hpi and persisted until early schizogony. In contrast, VAMP8 (Figure 1B) localized to the parasite only during the schizogony growth phase (24–40 hpi), while Stx7 (Figure 1C) showed weak recruitment at all time points compared to VAMP7 and VAMP8. None of the SNARE proteins were detected on the parasite at 48 hpi (Figure 1A–C).

To determine whether these SNARE candidates are involved earlier in parasite infection specifically in its establishment prior to 6 hpi, we assessed their recruitment to the PVM at earlier time points. We performed IFA at very early time points post-infection (0.5, 1, and 1.5 hpi; (Figure 1D–G)). Since Vti1B GFP tagging was unsuccessful, a monoclonal anti-Vti1B antibody was used. Vti1B and VAMP7 (Figure 1D,E) were detected on the PVM as early as 0.5 hpi, suggesting their involvement in initial establishment of parasite infection. However, VAMP8 and Stx7 (Figure 1F,G) were absent from the PVM at these early stages, indicating that they are likely recruited later to facilitate vesicle fusion events.

Given that SNARE recruitment to the PVM peaked at 24 hpi (Supplementary Figure S2A), we performed expansion microscopy on infected cells at this time point, confirming their strong colocalization with the parasite PVM (Figure 1H). In addition, the recruitment of host VAMP7 to the PVM was further examined using Stimulated Emission Depletion (STED) microscopy (Supplementary Figure S1C). To determine whether the recruitment of SNARE candidates to the PVM observed in HeLa cells also occurs in primary hepatocytes, the latter were isolated from mice and infected with *Plasmodium* sporozoites. As observed in HeLa cells, VAMP7, Vti1B, VAMP8, and Sxt7 were found to be recruited to the parasite PVM (Supplementary Figure S1D).

### 3.2. SNARE Proteins Colocalize with LAMP1-Positive Lysosomes and Regulate Lysosomal Homeostasis

**Figure 2:**
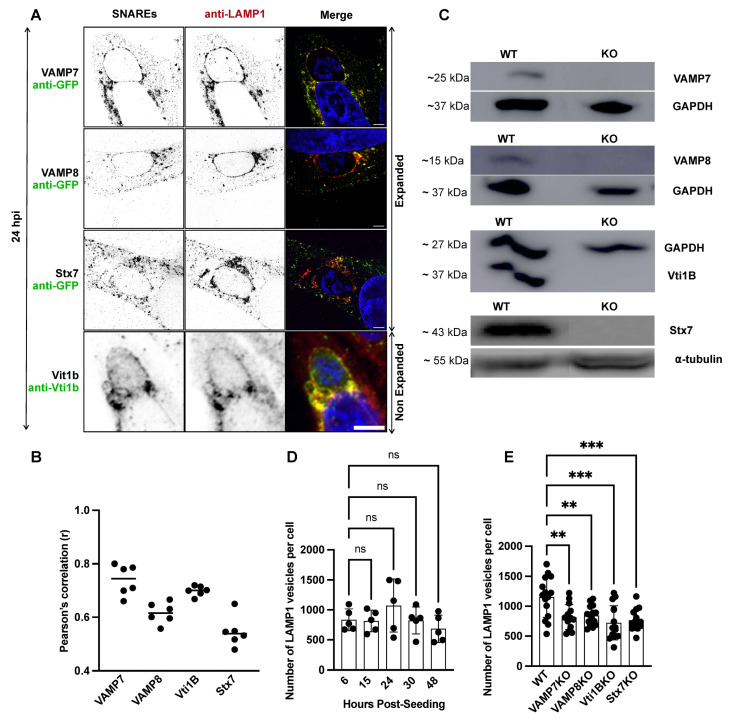
Role of SNARE proteins in LAMP1-positive lysosomal vesicle trafficking in *P. berghei*-infected HeLa cells. (**A**) Expansion microscopy (VAMP7, VAMP8, and Stx7) and standard IFA (Vti1B) confocal images showing colocalization of the SNARE proteins (green) with LAMP1 (red). Merged channels (yellow) highlight SNARE-LAMP1 positive vesicles association. Nuclei were stained with DAPI (blue). Scale bars: 10 µm. (**B**) Quantification of SNARE-LAMP1 colocalization using Pearson’s correlation coefficient (**B**): VAMP7 (r = 0.7), VAMP8 (r = 0.6), Vti1B (r = 0.9), Stx7 (r = 0.5). Each dot represents one cell; horizontal lines indicate mean values. (**C**) Immunoblot validation of SNARE knockout (KO) cell lines (VAMP7KO, VAMP8KO, Vti1BKO, Stx7KO) showing protein molecular weights. GAPDH (~37 kDa) and α-tubulin (~55 kDa) served as a loading control. (**D**) Quantification of LAMP1-positive vesicles using 3D and 4D IMARIS software (v10.2.0) over time in WT cells, with an average of 1073 LAMP1-positive vesicles per cell at 24 h post-seeding. Data represent mean ± SD; n = 25 cells (5 per experiment) per time point; ns = not significant. (**E**) Quantification of LAMP1-positive vesicles per cell at 24 H in WT (1152) vs. SNARE KO cell lines: VAMP7KO (817), VAMP8KO (845), Vti1BKO (722), Stx7KO (768). Data represent mean ± SD; n = 15 cells per cell line. Statistical significance was determined by one-way ANOVA with Dunnett’s multiple comparisons test (** *p* < 0.01, *** *p* < 0.001; ns, not significant).

Since SNARE candidates were found on the PVM during liver-stage infection, we investigated whether they colocalize with lysosomes and late endosomes using the lysosomal membrane marker LAMP1. HeLa cells were infected with *P. berghei* and fixed at 24 hpi. Cells were expanded and stained with anti-LAMP1 antibodies to label lysosomes and late endosomes, while SNARE proteins were detected using anti-GFP (for VAMP7, VAMP8, and Stx7) or anti-Vti1B antibodies (for Vti1B, standard IFA) (Figure 2A). Pearson’s correlation coefficient analyses revealed that VAMP7, VAMP8, and Vti1B colocalized with LAMP1, each showing a Pearson’s correlation coefficient (r) above 0.6. In contrast, Stx7 displayed lower colocalization, although its coefficient remained slightly above 0.5 (Figure 2B). The colocalization of SNARE proteins with both lysosomes and the PVM suggests they may play a crucial role in mediating lysosomal vesicle fusion with the PVM.

To explore the functional relevance of these SNARE proteins during liver-stage parasite development, we generated HeLa cell lines with individual knockouts (KOs) of VAMP7, VAMP8, Vti1B, and Stx7 using a CRISPR/Cas9 approach. Following generation by limiting dilution, clonal knockout cell lines were assessed for viability and proliferation. The knockout was found to have no adverse effect on these parameters. Successful knockout was subsequently confirmed by western blotting before further experimental use (Figure 2C, Supplementary Figure S3). To assess the impact of SNARE KO on lysosome and late endosome distribution and abundance within the cells, we quantified the number of LAMP1-positive vesicles in wild-type (WT) HeLa cells at multiple time points post-seeding (6 h, 15 h, 24 h, 30 h, and 48 h) (Figure 2D). Lysosome and endosome numbers remained consistent over time in WT cells. However, a significant reduction in LAMP1-positive vesicles was observed in all SNARE KO cell lines compared to WT at 24 h (Figure 2E), suggesting that the loss of these SNARE proteins disrupts lysosomal and endosomal vesicle homeostasis, potentially affecting both membrane fusion machinery and lysosomal content.

### 3.3. Effect of SNARE Protein Depletion on Parasite Infection and Survival

**Figure 3:**
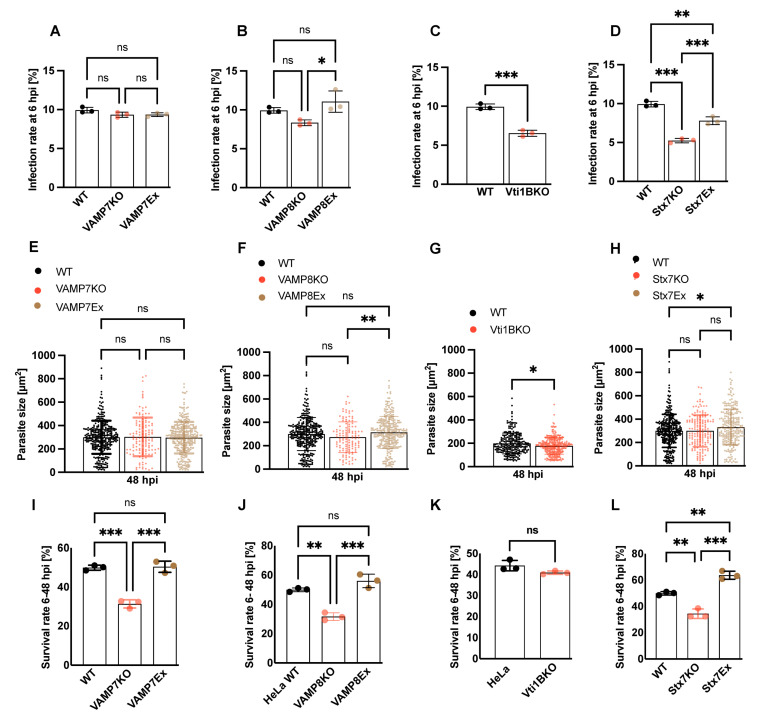
Role of SNARE candidates in *P. berghei* liver-stage infection and development in HeLa cells. (**A**–**D**) Infection rates at 6 hpi in WT HeLa cells and SNARE knockout (KO) or rescue (Ex) lines infected with *P. berghei* sporozoites. No significant difference was observed in VAMP7KO cells (9.3%) (**A**), whereas infection was significantly reduced in VAMP8KO (8.3%) (**B**), Vti1BKO (6.5%) (**C**), and Stx7KO (5.2%) (**D**) cells compared to WT (10%). Overexpression with the respective SNARE proteins partially restored infection rates. (**E**–**H**) Parasite size quantified at 48 hpi in WT and KO/Ex cell lines. No significant differences in parasite size were observed in the SNARE KO or Ex lines except for Vti1B. (**I**–**L**) Parasite survival rate (6–48 hpi) in WT and KO/Ex lines. Parasite survival was significantly reduced in VAMP7KO (**I**), VAMP8KO (**J**), and Stx7KO (**L**) cells, but not in Vti1BKO cells (**K**), indicating that these SNAREs are critical for parasite survival during liver-stage development. Data are presented as mean ± SD. Statistical significance was determined by one-way ANOVA with Dunnett’s multiple comparisons, appropriate post hoc tests, (**C**,**G**,**K**) and Student’s *t*-test. * *p* < 0.05, ** *p* < 0.01, *** *p* < 0.001; ns, not significant.

To investigate the role of SNARE candidates in parasite development, SNARE KO HeLa cells were infected with *P. berghei* sporozoites. At 6 hpi, parasites were counted, and the infection rate was calculated based on the number of sporozoites used for infection. VAMP7KO cells did not show any change in infection rate compared to control cells (Figure 3A), suggesting that VAMP7 is not required for parasite invasion, despite its strong recruitment to the parasite as early as 30 min after infection (Figure 1A, Video S1). In contrast, VAMP8KO, Vti1BKO, and Stx7KO cells exhibited a reduced infection rate compared to the control (Figure 3B–D). Parasite size was measured at 48 hpi across the KO cell lines. Although a trend of reduced parasite size was observed in all SNARE candidate KO cells, the differences were not statistically significant at any of the time points measured (Figure 3E–H). Notably, a significant reduction in parasite survival was observed in all KO cell lines compared to the control (Figure 3I,J,L), except for Vti1BKO cells (Figure 3K). These findings indicate a crucial role for specific SNARE proteins in supporting parasite invasion and survival during liver-stage development.

### 3.4. SNARE Protein Depletion Does Not Impair Lysosomal Recruitment to the PVM but Alters Vesicle Distribution

**Figure 4:**
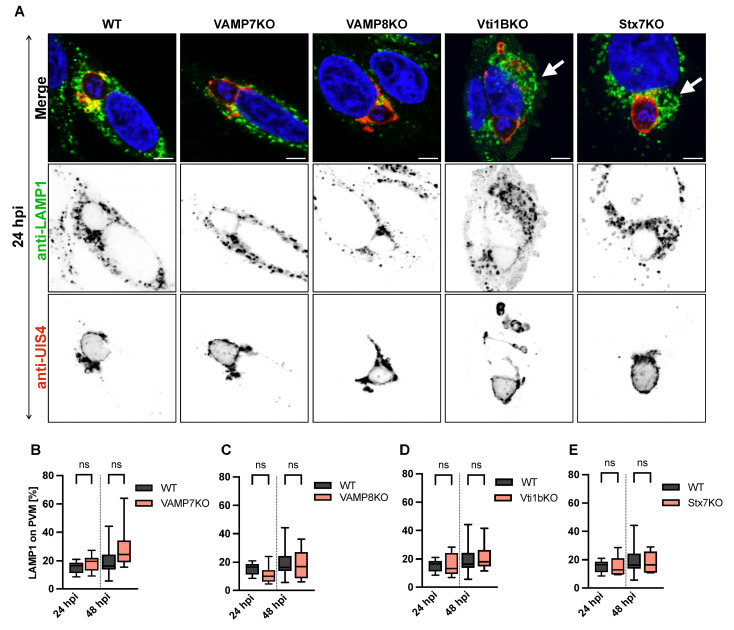
Impact of SNARE depletion on LAMP1-positive vesicle association with the *P. berghei* PVM and intracellular distribution. (**A**) Representative confocal microscopy images of HeLa cells infected with *P. berghei* sporozoites and fixed with 4% paraformaldehyde (PFA) at 24 hpi. Immunofluorescence staining was performed using anti-LAMP1 (lysosomes, green) and anti-UIS4 (PVM, red). Nuclei were counterstained with DAPI (blue). Note the altered vesicle distribution in KO cells, with LAMP1-positive vesicles showing perinuclear clustering (arrows in (**A**)) compared to the dispersed pattern in WT cells. Z-stack images were processed using IMARIS software for 3D/4D reconstruction: LAMP1-positive vesicles were quantified as isospots, while the PVM was rendered as an isosurface. The percentage of LAMP1 vesicles associated with the PVM was calculated based on isospots located within 0–0.5 µm of the PVM isosurface. (**B**–**E**) Quantification of LAMP1-positive vesicles in contact with the PVM (normalized to total LAMP1 vesicles per cell) at 24 and 48 hpi. No significant differences were observed between wild-type (WT) and VAMP7KO (**B**), VAMP8KO (**C**), Vti1BKO (**D**), or Stx7KO (**E**) cells. Scale bar: 5 µm. Data represent mean ± SD; n = 25 cells per condition. Statistical significance between time points and conditions was determined by one-way ANOVA with Dunnett’s multiple comparisons test; ns, not significant.

To determine whether depletion of specific SNARE proteins affects lysosomal fusion with the *P. berghei* PVM, we quantified the association of LAMP1-positive vesicles with the parasite’s PVM in SNARE KO HeLa cells using 3D and 4D reconstruction using the IMARIS software (v10.2.0) (Figure 4A–E). The number of LAMP1-positive vesicles on the PVM was normalized to the total number of LAMP1-positive vesicles per cell at 24 and 48 hpi. The quantification revealed no significant differences in vesicle recruitment between WT and VAMP7KO, VAMP8KO, Vti1BKO, or Stx7KO cells (Figure 4B–E). However, we observed a striking alteration in vesicle distribution. In WT cells, LAMP1-positive vesicles were dispersed throughout the cytoplasm, whereas in SNARE KO cells, they exhibited a more clustered polarized localization, accumulating near the perinuclear region (Figure 4A), as previously described [49,50,51]. This suggests that while SNARE depletion does not impair lysosomal recruitment to the PVM, it may disrupt vesicle trafficking or spatial organization.

### 3.5. Double Knockout of SNARE Genes Impairs Parasite Development and Lysosome–PVM Fusion

**Figure 5:**
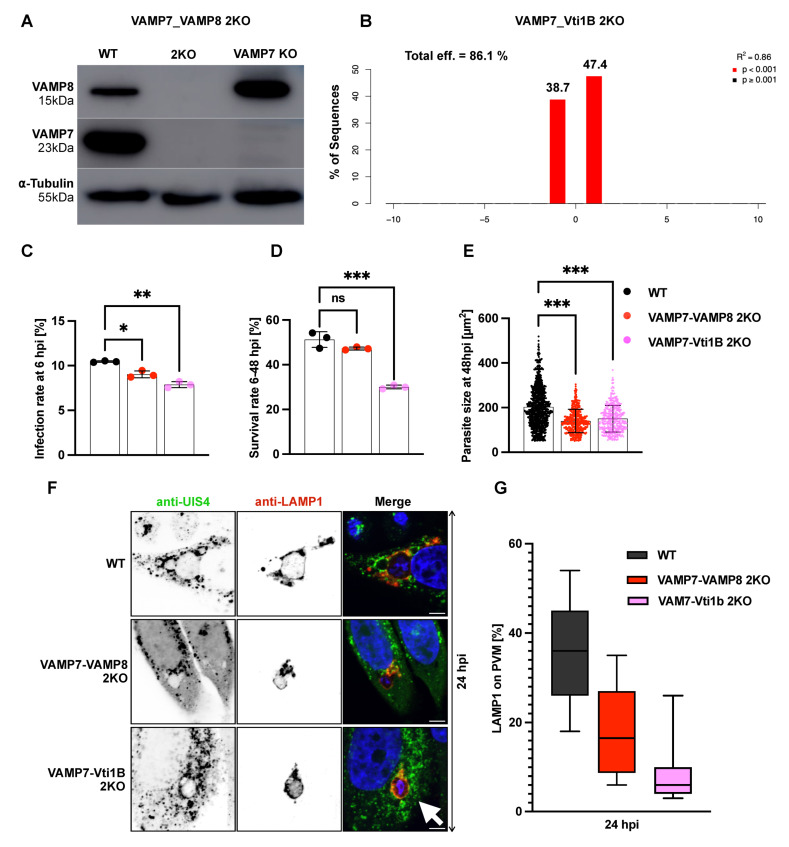
Double knockout of SNARE candidates impairs *P. berghei* liver-stage development. (**A**,**B**) Generation and validation of VAMP7-VAMP8 and VAMP7-Vti1B 2KO HeLa cell lines. (**A**) Western blot analysis confirms efficient depletion of VAMP7 and VAMP8 proteins in VAMP7-VAMP8 2KO cells; (**B**) validation of Vti1B knockout in VAMP7KO cells. Due to their similar molecular weights (~23–27 kDa), confirmation of the VAMP7-Vti1B 2KO was performed using TIDE (Tracking of Indels by Decomposition). The TIDE plot confirms successful Vti1B knockout in VAMP7KO cells, with a total editing efficiency of 86.1%. (**C**) Infection rates of *P. berghei* sporozoites at 6 h post-infection (hpi). Both 2KO lines showed significantly reduced infection (VAMP7-VAMP8 2KO: 9%; VAMP7-Vti1B 2KO: 7.8%) compared to wild-type (WT) cells (10.5%). (**D**) Parasite survival (6–48 hpi,) was significantly decreased in VAMP7-Vti1B 2KO cells (30%) compared to WT (51.2%), while VAMP7-VAMP8 2KO cells showed a non-significant reduction (47.2%). Each experiment was conducted in triplicate. (**E**) Parasite size quantification at 48 hpi. Both 2KO lines exhibited impaired growth: VAMP7-VAMP8 2KO: 66 µm^2^ (24 hpi), 140 µm^2^ (48 hpi); VAMP7-Vti1B 2KO: 67 µm^2^ (24 hpi), 150 µm^2^ (48 hpi) compared to WT: 73 µm^2^ (24 hpi), 203 µm^2^ (48 hpi). Each dot represent a single measured parasite (**C**–**E**), WT: black bots, VAMP7-VAMP8 2KO: red dots and VAMP7-Vti1B: magenta dots. (**F**) Confocal microscopy images of infected cells at 24 hpi stained with anti-UIS4 (PVM, red) and anti-LAMP1 (lysosomes, green). VAMP7-VAMP8 2KO cells showed reduced LAMP1 signal, while VAMP7-Vti1B 2KO cells exhibited perinuclear accumulation of LAMP1-positive vesicles near parasites (arrow). Nuclei were counterstained with DAPI (blue). Scale bar: 5 µm. (**G**) Quantification of LAMP1-positive vesicle association with the PVM at 24 hpi using 3D/4D IMARIS. Both, 2KO lines showed significantly reduced attachment: VAMP7-VAMP8 2KO: 18%; VAMP7-Vti1B 2KO: 9.1% compared to WT: 36%. Data represent mean ± SD. Statistical significance was determined by one-way ANOVA with appropriate post hoc tests: * *p* < 0.05, ** *p* < 0.01, *** *p* < 0.001; ns, not significant.

Given the known functional redundancy among SNARE proteins and the frequent co-expression of VAMP7, VAMP8, and Vti1B, we investigated whether combined depletion would enhance phenotypic effects on *P. berghei* liver-stage development. Since single SNARE KOs did not significantly impair parasite growth or lysosome–PVM fusion, we generated double knockout (2KO) HeLa cell lines using CRISPR/Cas9. Specifically, we generated two combinations: VAMP8 depletion in a VAMP7KO background (VAMP7-VAMP8 2KO) and Vti1B depletion in a VAMP7KO background (VAMP7-Vti1B 2KO). These double knockouts had no adverse effect on host cell viability or proliferation. In contrast, any 2KO combination involving Stx7 proved deleterious to host cells, abolishing both proliferation and viability. This finding underscores the essential role of Stx7 in mediating homotypic fusion and SNARE complex formation. Clonal lines of VAMP7-VAMP8 2KO were validated by western blotting (Figure 5A, Supplementary Figure S3). Due to their similar molecular weights (~23–27 kDa), the VAMP7-Vti1B 2KO was confirmed not by western blot, but by TIDE (Tracking of Indels by Decomposition) genotyping (Figure 5B). Both 2KO cell lines exhibited significantly reduced infection rates compared to the WT control (Figure 5C) and VAMP7-Vti1B impaired parasite survival between 6 and 48 hpi (Figure 5D). Parasite growth was consistently affected at 48 hpi, with significant reduction in parasite size in both 2KO lines (Figure 5E), demonstrating that these SNARE combinations are crucial for supporting parasite development.

To examine lysosomal fusion with the PVM, we performed immunofluorescence assays labeling lysosomes with anti-LAMP1 antibodies and the PVM using anti-UIS4 antibodies (Figure 5F). VAMP7-VAMP8 2KO cells showed an overall reduction in LAMP1-positive vesicles, while VAMP7-Vti1B 2KO cells exhibited perinuclear accumulation of LAMP1-positive vesicles near parasites, suggesting distinct trafficking defects in each 2KO line. Quantitative 3D/4D analysis using IMARIS revealed significantly reduced association of LAMP1-positive vesicles with the PVM at 24 hpi in both 2KO lines compared to controls (Figure 5G). These results demonstrate that combined SNARE depletion disrupts both lysosome–PVM fusion and compromises parasite development during the liver-stage.

### 3.6. Triple SNARE Knockout Further Impairs Parasite Liver-Stage Development and Lysosome–PVM Interaction

Previous experiments demonstrated that single SNARE KOs did not significantly affect *P. berghei* liver-stage development, suggesting potential functional redundancy (Figure 3 and Figure 5). Notably, the 2KO of VAMP7 and Vti1B resulted in a significant reduction in parasite infection, growth, and survival, whereas the VAMP7–VAMP8 2KO primarily impaired parasite infection and growth, with no significant effect on survival. These findings support the idea that SNARE proteins act redundantly and potentially compensate for each other in vesicle trafficking pathways relevant to parasite development.

**Figure 6:**
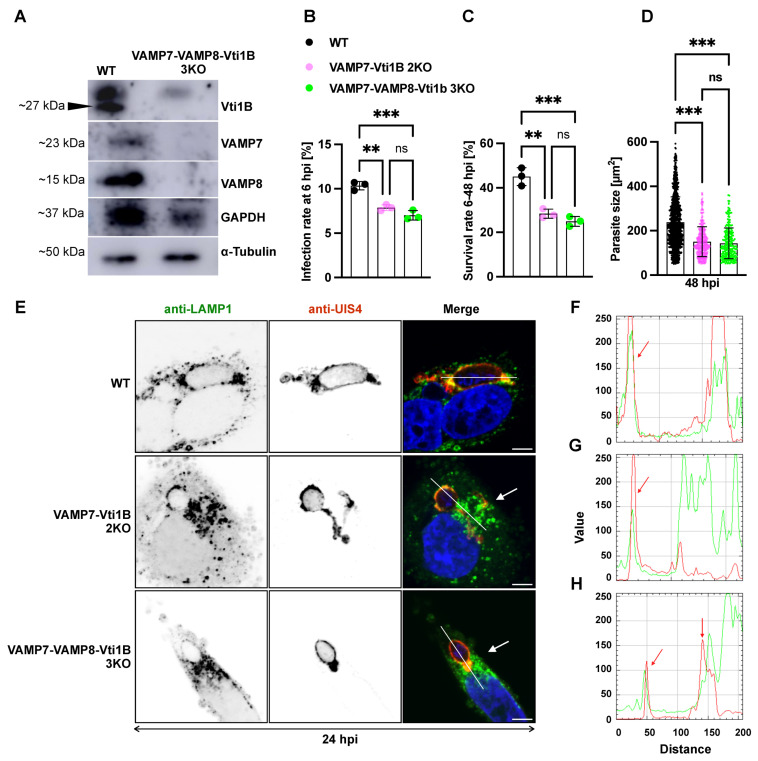
Triple SNARE knockout exacerbates impairment of *P. berghei* liver-stage development and lysosome–PVM interaction. (**A**) Western blot validation of VAMP7-VAMP8-Vti1B triple knockout (3KO) HeLa cells. Efficient depletion of all three SNARE proteins in the 3KO cell line is shown compared to wild-type (WT) controls. Vti1B (~27 kDa) deletion was first confirmed in a VAMP7-VAMP8 double knockout (2KO) background, with α-Tubulin and GAPDH serving as loading controls. The subsequent absence of VAMP7 (~23–25 kDa) and VAMP8 (~15 kDa) in the 3KO line was also verified, using GAPDH as a loading control, prior to further experiments. (**B**) Infection rates at 6 hpi showing significant reduction in VAMP7-Vti1B 2KO and VAMP7-Vti1B-VAMP8 3KO compared to the WT. No significant difference was observed between 2KO and 3KO. (**C**) Parasite survival (6–48 hpi) was significantly decreased in both 2KO and 3KO compared to the WT, with no additional significant effect in 3KO versus 2KO. (**D**) Parasite size quantification at 48 hpi (significant impairment in both genotypes). No significant difference was observed between 2KO and 3KO at any timepoint. (**E**) Immunofluorescence confocal images at 24 hpi showing LAMP1 (lysosomes, green), UIS4 (PVM, red), and DAPI (nuclei, blue). Note: perinuclear LAMP1 accumulation in 2KO and 3KO versus dispersed distribution in WT (Arrow). (**F**–**H**) Fluorescence intensity profiles across infected cells following the white line in the cell (PVM = red line and LAMP1 signal = green lines) demonstrating strong LAMP1-UIS4 colocalization at PVM in WT ((**F**), red arrow), partial colocalization in VAMP7-Vti1B 2KO, and (**G**) nearly absent colocalization in 3KO ((**H**), red arrow). Lysosome accumulation within the cytoplasm was also visible as a broadening of the curves (**G**–**H**) Scale bars: 5 µm (**B**–**D**). Data represent mean ± SD; statistical significance (one-way ANOVA with Dunnett’s test): ** *p* < 0.01, *** *p* < 0.001; ns, not significant.

To test whether a more extensive disruption of SNARE function would further impair liver-stage development, we generated a triple knockout (3KO) cell line by depleting VAMP8 in the VAMP7–Vti1B 2KO background using CRISPR/Cas9. The resulting VAMP7–Vti1B–VAMP8 3KO HeLa cells were cloned and validated by western blot (Figure 6A, Supplementary Figure S3). The triple knockout (3KO) generated clonal lines with distinct phenotypes. While some clones exhibited no defects in viability or morphology, others displayed a slender cell shape and reduced proliferation (Supplementary Figure S2B–E). Clones unaffected in viability were selected for subsequent experiments (Supplementary Figure S2B–E).

Infection of the 3KO cells with *P. berghei* sporozoites revealed a significant reduction in infection rate compared to control cells (Figure 6B). Parasite survival was also significantly impaired in the 3KO line relative to WT (Figure 6C). However, when compared to the VAMP7–Vti1B 2KO, the additional loss of VAMP8 resulted in only a slight further reduction in infection and survival, which did not reach statistical significance. To assess the effect on parasite growth, parasite size was measured at 48 hpi (Figure 6D). The parasite growth was significantly reduced at 48 hpi in the 3KO compared to WT controls. Size comparisons as well as infection and survival rates did not differ between 2KO and 3KO parasites.

To determine whether the 3KO had a stronger effect on host lysosome association with the PVM, we performed IFA in both the 2KO and 3KO cells, staining lysosomes with anti-LAMP1 antibodies and the parasite PVM with anti-UIS4 antibodies (Figure 6E). As previously observed in the VAMP7–Vti1B 2KO, LAMP1-positive vesicles accumulated near the perinuclear region in both 2KO and 3KO cells, in contrast to their more dispersed cytoplasmic distribution in WT cells.

To further evaluate LAMP1 distribution and colocalization with the PVM, fluorescence intensity profiles were generated across the host cell cytoplasm and the PVM using the RGB profiler tool in FIJI (Figure 6F–H). In WT cells, LAMP1 signals strongly colocalized with UIS4 at the PVM (Figure 6F). Partial colocalization was observed in the VAMP7–Vti1B 2KO (Figure 6G), whereas in the 3KO cells, colocalization was nearly absent, indicating a disruption of the lysosome–PVM interaction in the absence of all three SNARE candidates (Figure 6H).

These findings suggest that although the additional loss of VAMP8 in the VAMP7–Vti1B 2KO background does not substantially worsen parasite infection or survival, it further impairs the lysosome–PVM association, highlighting a cumulative role for SNARE proteins in maintaining host vesicles–parasite membrane fusion during liver-stage development.

### 3.7. Proposed Model of SNARE-Mediated Host Lysosome–PVM Interactions During Plasmodium Berghei Liver-Stage Development

**Figure 7:**
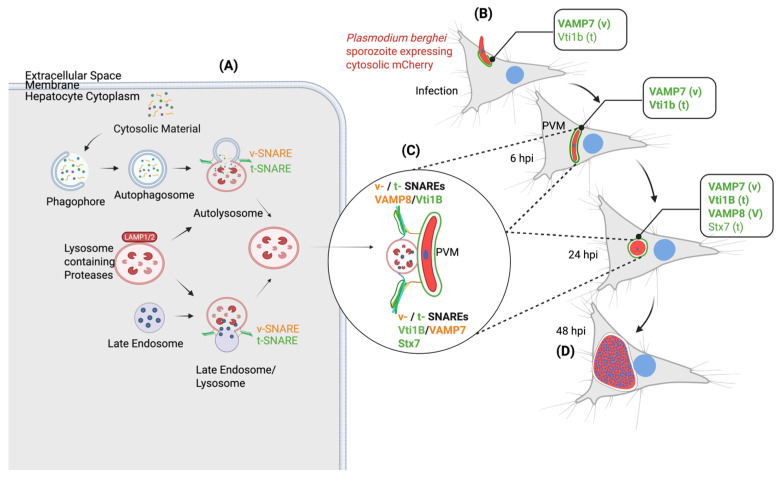
SNARE-mediated lysosome–PVM fusion supports *Plasmodium* liver-stage development. This schematic illustrates how host SNARE proteins mediate membrane fusion between host lysosomal vesicles and the *Plasmodium* PVM, facilitating parasite development in hepatocytes. (**A**) In uninfected hepatocytes, cytosolic material is degraded through the endolysosomal and autophagic pathways. Fusion events between phagophores, autophagosomes, late endosomes, and lysosomes are orchestrated by specific v-SNAREs (vesicle SNAREs, shown in orange) and t-SNAREs (target membrane SNAREs, shown in green). LAMP1 serves as a lysosomal marker. (**B**–**D**) Upon infection with *P. berghei* sporozoites expressing cytosolic mCherry, the parasite resides within a PVM and undergoes liver-stage development. (**B**) At early stages (6 hpi), host v-SNARE VAMP7 is recruited to the PVM formed through invagination of the host plasma membrane, bringing with it the associated t-SNARE Vti1B. (**C**) As the infection progresses (24 hpi), additional host SNAREs including VAMP8 (v) and Stx7 (t) are found on LAMP1 positive lysosomal vesicles that dock at the PVM, likely facilitating membrane fusion. This delivers lysosome-associated cargo (cytosolic material) to support schizogony (**D**). Together, these observations support a model where *Plasmodium* hijacks the host’s SNARE-dependent lysosomal trafficking machinery to import host-derived nutrients across the PVM. This process is essential for the parasite’s intracellular growth and successful liver-stage development. v = v-SNARE; t = t-SNARE. Created with BioRender.com.

The schematic in Figure 7 illustrates the molecular mechanism by which host SNARE proteins facilitate lysosomal vesicle fusion with the *Plasmodium* PVM, a process critical for successful liver-stage development. In uninfected hepatocytes, SNARE proteins mediate membrane fusion events essential for endolysosomal trafficking (Figure 7A). v-SNAREs and t-SNAREs localize to distinct compartments, coordinating vesicle docking and fusion involved in autophagosome–lysosome and endosome–lysosome formation. LAMP1 serves as a lysosomal membrane marker. Upon invasion by a *P. berghei* mCherry-expressing sporozoite, the parasite becomes enclosed in the PVM derived from host plasma membrane invagination (Figure 7B). Host SNARE proteins are recruited to this interface in a temporally regulated manner. At early time points (from invasion to 6 hpi), VAMP7s (host v-SNAREs) are constitutively present at the PVM (green), likely incorporated just after host membrane invagination. The VAMP7s on the PVM mediate lysosomes fusion to the PVM with the help of the host Vti1B. As infection progresses (by 24 hpi), additional SNARE components, namely VAMP8 and Stx7 (from host lysosomal vesicles, orange), are recruited and docked at the PVM (Figure 7C). These SNARE complexes facilitate the targeted fusion of host lysosme with the PVM. This fusion process is hypothesized to enable the delivery of lysosome-associated cargo, including cytosolic material and possibly nutrients, which the parasite utilizes to support its rapid growth and replication during schizogony (Figure 7D), culminating in merozoite formation. Functional evidence from SNARE knockout experiments (Figure 3, Figure 4, Figure 5 and Figure 6) supports this model. Disruption of specific SNARE components impairs the lysosome–PVM association, reduces parasite size and survival, and ultimately compromises liver-stage development. These findings underscore the role of membrane-specific SNARE complexes in mediating nutrient acquisition from host lysosomal vesicles, an essential step for the parasite’s intracellular development.

## 4. Discussion

During *Plasmodium* liver infection, the parasite exhibits one of the fastest growth rates observed in eukaryotic cells, driven by parasite scavenging of host cell nutrients. Recent studies demonstrate that host lysosomal and late endosomal vesicles fuse with the PVM during the liver-stage [11,12,13,15]. While these vesicles are known to be critical for parasite development, the molecular mechanisms by which the parasite targets and manipulates host organelles to acquire nutrients remain poorly understood. Given the fundamental role of SNARE proteins in overcoming the energetic barrier to membrane fusion, our study investigated whether host SNARE machinery mediates lysosome–PVM fusion to support parasite development. The PVM is a critical interface for *Plasmodium* liver-stage development, uniquely adapted to recruit host-derived vesicles while minimizing parasite protein exposure to the host cytoplasm [52]. The liver stage PVM may actively hijack host trafficking pathways through parasite-encoded SNAREs or lipid-based signals. Transcriptomic analyses revealed the upregulation of *Plasmodium* SNARE homologs during liver infection [53], including Q-SNAREs (Stx1) predicted to localize to the parasite plasma membrane [54]. However, their direct presence at the PVM remains undetected [25,55], suggesting alternative mechanisms where the parasite hijacks host-derived SNAREs for vesicle recruitment. Using the CRISPR/Cas9 KO approach and advanced microscopy tools, our study elucidates how host SNARE proteins mediate vesicular trafficking between lysosomes and the PVM, revealing a host-dependent nutrient acquisition pathway essential for parasite development.

Our data support a two-phase model of SNARE involvement at the PVM. Early recruitment of VAMP7 and Vti1B occurs shortly after invasion and likely primes the PVM for subsequent vesicle interactions, whereas later recruitment of VAMP8 and Stx7 coincides with the onset of rapid parasite growth during schizogony. The remarkable speed of this process within 30 min mirrors rapid host machinery co-option seen in other pathogens. *Salmonella* similarly recruits LC3-positive autophagic structures within 10 min of infection [56]. This model is supported by the delayed recruitment (24 hpi) of VAMP8, a v-SNARE participating in lysosomal fusion complexes with Vti1B and Stx7 coinciding with the onset of rapid parasite nuclear replication and biomass expansion [57,58]. The t-SNARE Stx7 shows weak PVM association at 6 hpi but becomes more prominent by 24 hpi (Figure 1 and Supplementary Figure S2), consistent with its known role in late endosomal/lysosomal fusion when partnered with Vti1B and VAMP8. Functional genetic studies demonstrated significant redundancy among SNARE proteins [31,59,60,61]. Functional redundancy among SNARE proteins likely explains why single knockouts had limited phenotypic impact, whereas combinatorial depletion revealed pronounced defects. In particular, disruption of Vti1B-containing complexes severely impaired parasite survival and the lysosome–PVM association, identifying Vti1B as a central node in this pathway. The failure to generate viable Stx7-containing combinatorial knockouts further underscores the essential role of this SNARE in core lysosomal and autophagic processes required for host cell viability.

These findings integrate well with existing models of host–parasite interactions at the PVM. During liver-stage infection, parasites activate the PAAR response and recruit components such as the V-type ATPase to the PVM, processes traditionally associated with parasite elimination [35,62,63]. Our data instead support a model in which *Plasmodium* selectively exploits lysosomal fusion to access host-derived resources while avoiding destructive acidification. This balance is likely facilitated by PVM properties, including pores that allow nutrient exchange while dissipating proton gradients.

Lysosomes thus represent not only degradative organelles but also reservoirs of lipids, amino acids, and metabolites that can be repurposed to support parasite growth. By hijacking host SNARE-mediated trafficking, *Plasmodium* converts a potentially hostile compartment into a supportive source for its fast intracellular development.

Our SNARE-related findings complement these understandings, as vesicle trafficking defects in knockout cells might disrupt these processes. The potential interaction between our identified SNARE candidates and previously identified Rab GTPases provides additional mechanistic insight. *Plasmodium*-essential Rab5b and Rab11a orchestrate vesicle sorting, transport, and fusion, processes critical for parasite development [6,64]. The convergence of SNARE-mediated fusion with Rab-regulated vesicle trafficking suggests coordinated host pathway subversion, where *Plasmodium* simultaneously manipulates multiple aspects of vesicle dynamics to support intracellular growth.

## 5. Conclusions

In conclusion, this study identifies VAMP7, VAMP8, Vti1B, and Stx7 as host SNARE proteins hijacked by *Plasmodium* during liver-stage infection. Previous work has shown that host endosomal and lysosomal vesicles fuse with the parasite’s PVM, and that this fusion is essential for cholesterol sequestration and nutrient uptake. Moreover, disrupting this process reduces parasite burden in vivo during *Plasmodium* liver-stage development. Here, using cutting-edge microscopy and gene-editing tools, we demonstrate that *Plasmodium* exploits sequential SNARE interactions to potentially redirect lysosomal cargo including lipids and metabolites to the PVM. This work serves as a foundation for future research on host–pathogen interactions during the liver-stage infection.

Future work should focus on identifying the specific cargo delivered by these vesicles through proteomic and metabolomic analyses, and on validating the implicated mechanisms in primary human hepatocytes and in vivo models. In addition, biochemical membrane fusion assays are needed to provide more definitive validation of the SNARE-mediated fusion events. Although no specific, side-effect-free SNARE-targeting drugs are currently available to directly validate our findings in vitro, subsequent studies should prioritize the use of in vivo models to assess the therapeutic potential of targeting this pathway. This study not only advances our understanding of malaria liver-stage biology but also provides a paradigm for how intracellular pathogens exploit conserved membrane trafficking pathways.

## Figures and Tables

**Table 1:** SNARE protein localization at the PVM during liver stage infection.

SNARE Protein	Subcellular Localization [24]	Construct	^(^*^)^ Construct Origin	^(†)^ Association with PVM
SNAP29	Cytosolic, PM, EE, RE	GFP-SNAP29	Lab	−
		Flag-SNAP29	[45]	
Stx3	PM, Phagosomes	Antibody	Lab	+
Stx6	PM, EE, RE, LE, Golgi	GFP-Stx6	Lab	+
		3xFlag-Stx6	[46]	
Stx7	PM, EE, RE, LE, Lysosomes	Stx7-GFP	Lab	++
		Stx7-Myc	Gift by J. Brummel	
Stx8	EE, RE, LE, Lysosomes	GFP-Stx8	Lab	+
Stx17	Phagosomes, Autophagosomes	GFP-Stx17	Lab	+
		Flag-Stx17	[45]	
Stx11	PM, EE, RE, LE, Lysosomes, TGN	GFP-Stx11	Lab	−
Vti1b	EE, LE, Lysosomes, Golgi	antibody	Lab	+++
		Flag-Vti1b	[45]	
VAMP3	EE, RE, Phagosomes	GFP-VAMP3	Lab	−
VAMP7	PM, EE, LE, Lysosomes, Autophagosomes	GFP-VAMP7	Lab [47]	+++
VAMP8	PM, EE, RE, LE, Lysosomes, TGN	VAMP8-GFP	Lab	+++
		VAMP8-mCherry	[48]	

^(†)^ Association of SNARE constructs with the PVM was broadly categorized into − (no), + (weak), ++ (strong), +++ (very strong) through immunofluorescence analysis. ^(^*^)^ The origin of the plasmid construct. Lab indicates that plasmids were cloned by the authors. One million HeLa cells were transfected with 1 µg of plasmid DNA (construct). Following overnight incubation, the cells were infected with *Pb*mCherry sporozoites. At 24 hpi, cells and parasites were fixed with 4% PFA and immunostained with primary antibodies targeting the SNARE fusion protein (anti-GFP, anti-Flag) as well as the PVM. Antibodies against Vti1b and Stx3 were also used for detection, as described in the Methods Section 2. Stx, syntaxin; SNAP, synaptosomal-associated protein; VAMP, Vesicular-Associated Membrane Protein; Vti, Vesicle Transport Through Interaction With T-SNAREs; PM, plasma membrane; EE, early endosomes; RE, recycling endosomes; LE, late endosomes; TGN, trans-Golgi network; GFP, green fluorescent protein; PVM, parasitophorous vacuole membrane.

## Data Availability

The original contributions presented in this study are included in the article/Supplementary Materials. Further inquiries can be directed to the corresponding authors.

## Supplementary Materials

The following supporting information can be downloaded at https://www.mdpi.com/article/10.3390/cells15070584/s1: Videos S1–S3. Live imaging of *P. berghei* infected HeLa cells expressing VAMP7, VAMP8 and Stx7 GFP-tagged SNARE proteins. Time-lapse bright field fluorescence microscopy from 6 hpi shows the localization and dynamics of GFP-tagged host SNARE proteins during intracellular parasite development. Supplementary Figure S1. Recruitment of lysosomal and SNARE VAMP7 proteins to the *P. berghei* PVM. (A) PS-ExM of infected cells stained for the lysosomal marker LAMP1 (green) and the PVM marker UIS4 (red) at 24 hpi. The merged image with DAPI (blue) highlights host and parasite nuclei. Selected regions of interest (ROI 1–4) illustrate areas of close apposition reflecting possible membrane fusion between LAMP1-positive vesicles and the UIS4-positive PVM. Line intensity profiles across the indicated regions (yellow and magenta dashed lines) show the spatial distribution of LAMP1 (green) and UIS4 (red) signals, highlighting partial overlap or juxtaposition between host lysosomal structures and the PVM. Scale bars: 5 µm (overview) and 1 µm (insets). (B) Quantification of fluorescence intensity profiles corresponding to the ROIs in panel A. Plots show normalized fluorescence intensities for LAMP1 (green) and UIS4 (red) along the indicated line scans, illustrating the relative spatial relationship between host lysosomal compartments and the PVM. (C) High-resolution imaging using STED microscopy of infected cells stained with anti-UIS4 (magenta) and anti-GFP (green) at 24 hpi. The merged overview shows GFP-positive host vesicular structures surrounding and fusing with the UIS4-positive PVM. Insets (ROI 1 and ROI 2) highlight regions of vesicle accumulation adjacent to the PVM, suggesting vesicle docking or fusion sites. Scale bars: 3 µm (overview) and 1 µm (insets). (D) Localization of selected SNARE proteins relative to the PVM in infected primary hepatocytes at 24 hpi. Cells were stained with antibodies against VAMP7, Vti1B, VAMP8, and Syntaxin 7 (Stx7) (green) together with UIS4 (red). Merged images (with DAPI in blue) show recruitment of specific host SNARE proteins to the vicinity of the PVM, suggesting potential involvement of host vesicle fusion machinery during liver-stage infection. Supplementary Figure S2. (A) Localization of host SNARE proteins at the PVM during liver-stage infection. Colocalization of SNARE proteins (anti-Vti1B/anti-GFP) with the PVM marker UIS4, visualized by standard immunofluorescence assay. Merged channels (yellow) highlight PVM association. Nuclei were counterstained with DAPI (blue). Scale bars: 5 μm. (B-E) Phenotypic variation in triple knockout (3KO) clonal lines. Representative images (400× magnification) illustrate the phenotypic spectrum among 3KO clones following antibiotic selection. (Left to right): HeLa WT control; HeLa WT with antibiotics; Clone 1 (Cl1) with antibiotics; Clone 2 (Cl2) with antibiotics. (B) HeLa WT: Healthy, adherent cells with normal morphology. (C) HeLa WT + antibiotics: Complete cytotoxicity, evidenced by cell rounding, shrinkage, and loss of adhesion. (D) Cl1 + antibiotics: Heterogeneous population containing both dead, shrunken cells and viable cells with aberrant (star-shaped) morphology. (E) Cl2 + antibiotics: Predominantly healthy, adherent cells with minimal cytotoxicity. Selected for subsequent experiments. Supplementary Figure S3. Raw western blot images.

## Abbreviations

The following abbreviations are used in this manuscript:

*P. berghei*	*Plasmodium berghei*
PV	Parasitophorous Vacuole
PVM	Parasitophorous Vacuole Membrane
KO	Knock Out
SNARE	Soluble N-ethylmaleimide-sensitive factor attachment protein receptors
LAMP1	Lysosome-Associated Membrane Protein 1
TIDE	Tracking of Indels by Decomposition
VAMP	Vesicle-Associated Membrane Protein
VAMP7	Vesicle-Associated Membrane Protein 7
VAMP8	Vesicle-Associated Membrane Protein 8
Vti1B	Vesicle Transport Through Interaction With T-SNAREs 1B
Stx	Syntaxin
Stx7	Syntaxin 7
WB	Western blot
GAPDH	Glyceraldehyde-3-phosphate dehydrogenase
hpi	hour post infection
IFA	Immunofluorescence assay
GFP	Green fluorescence protein
ROI	Region of Interest
STED	Stimulated Emission Depletion
UIS4	Upregulated in Infectious Sporozoites 4
DAPI	4′,6-diamidino-2-phenylindole
PS-ExM	Pre-staining Expansion Microscopy
