# Supplementary material for: Host SNARE Proteins Mediate Lysosome and PVM Fusion to Support Plasmodium Liver Infection
Source: Cells. 2026 Mar 25;15(7):584. doi: 10.3390/cells15070584 (PMC13072298; doi:10.3390/cells15070584)

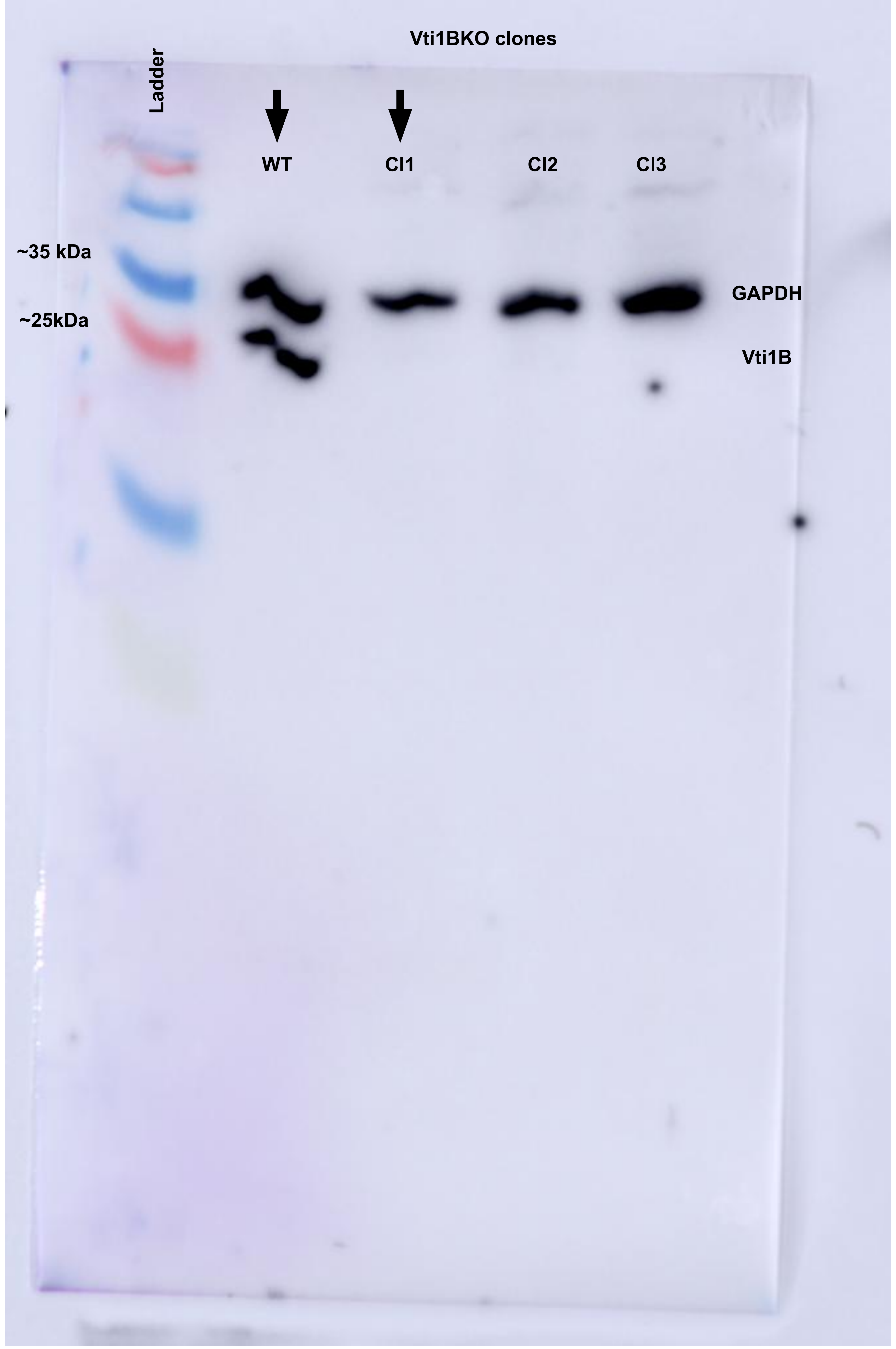

Vti1b KO clones WB

Used in the publication

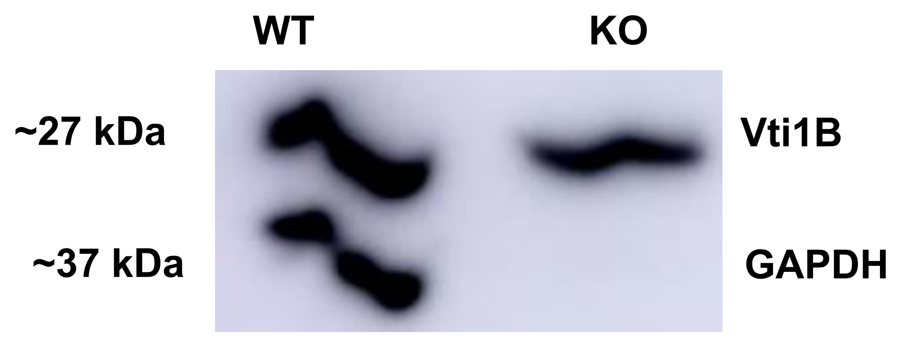

VAMP7 KO clones WB

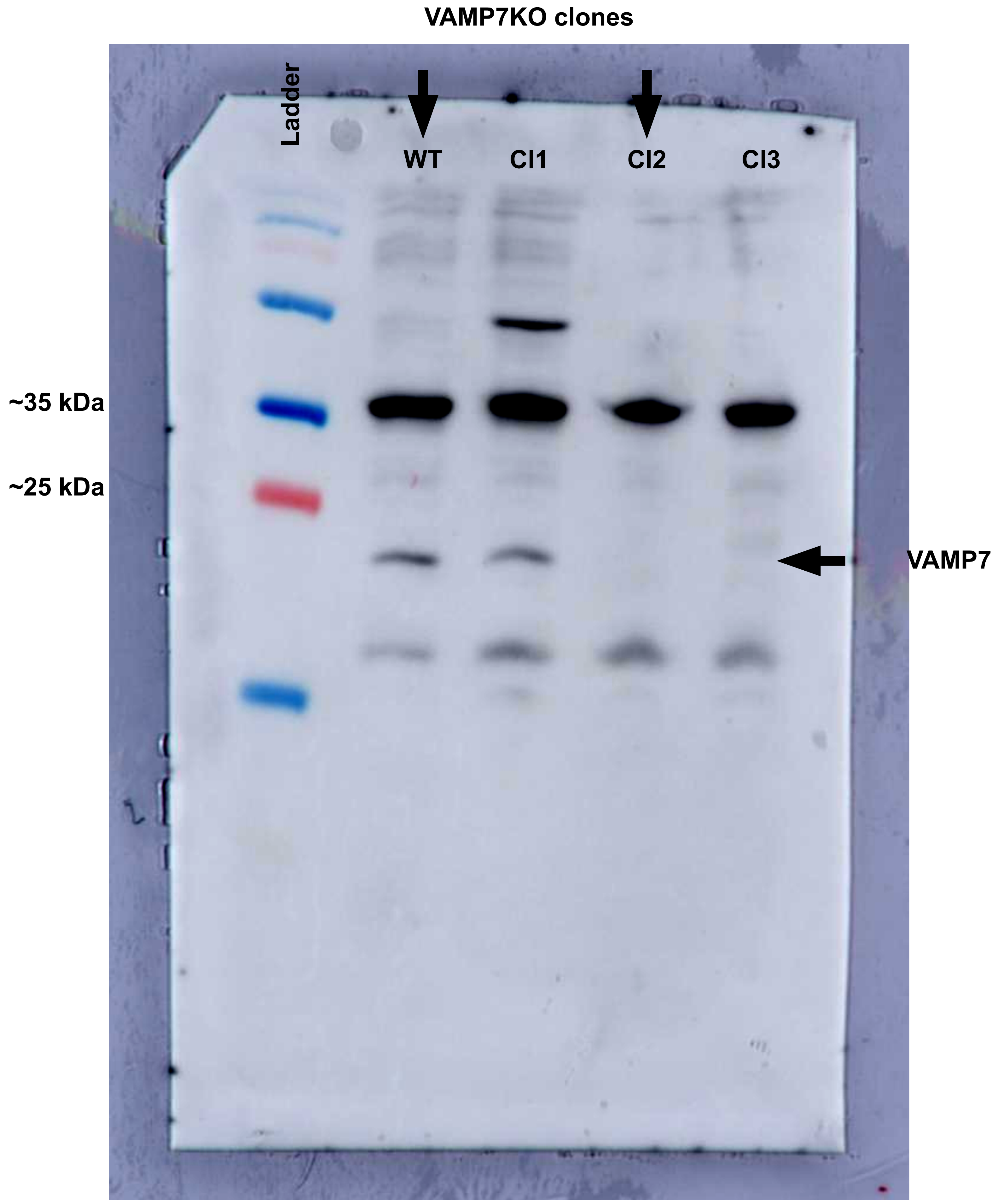

Used in the publication

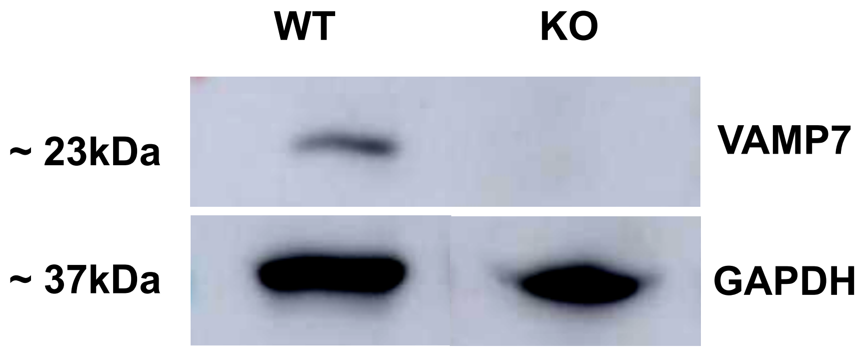

VAMP8 KO clones WB

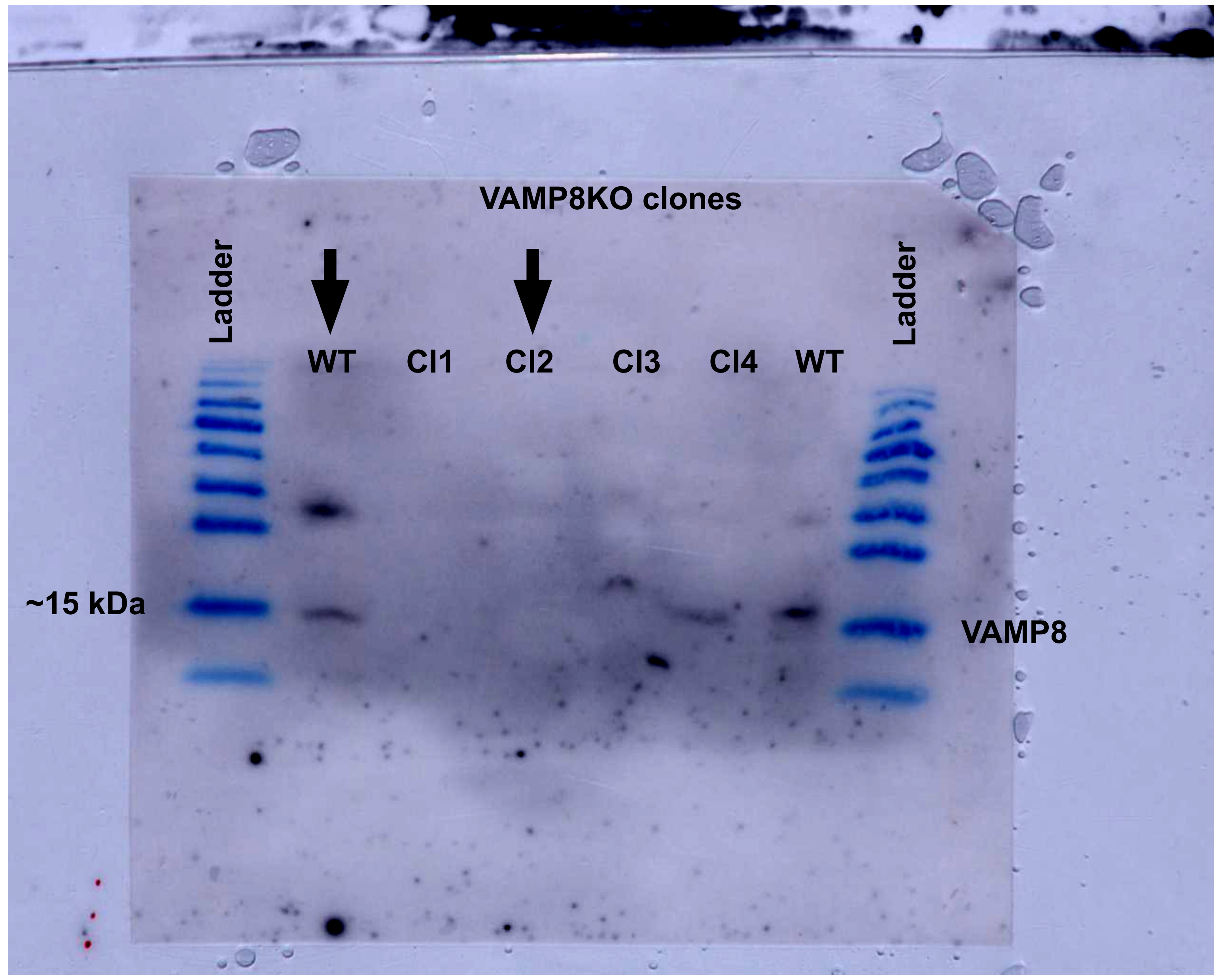

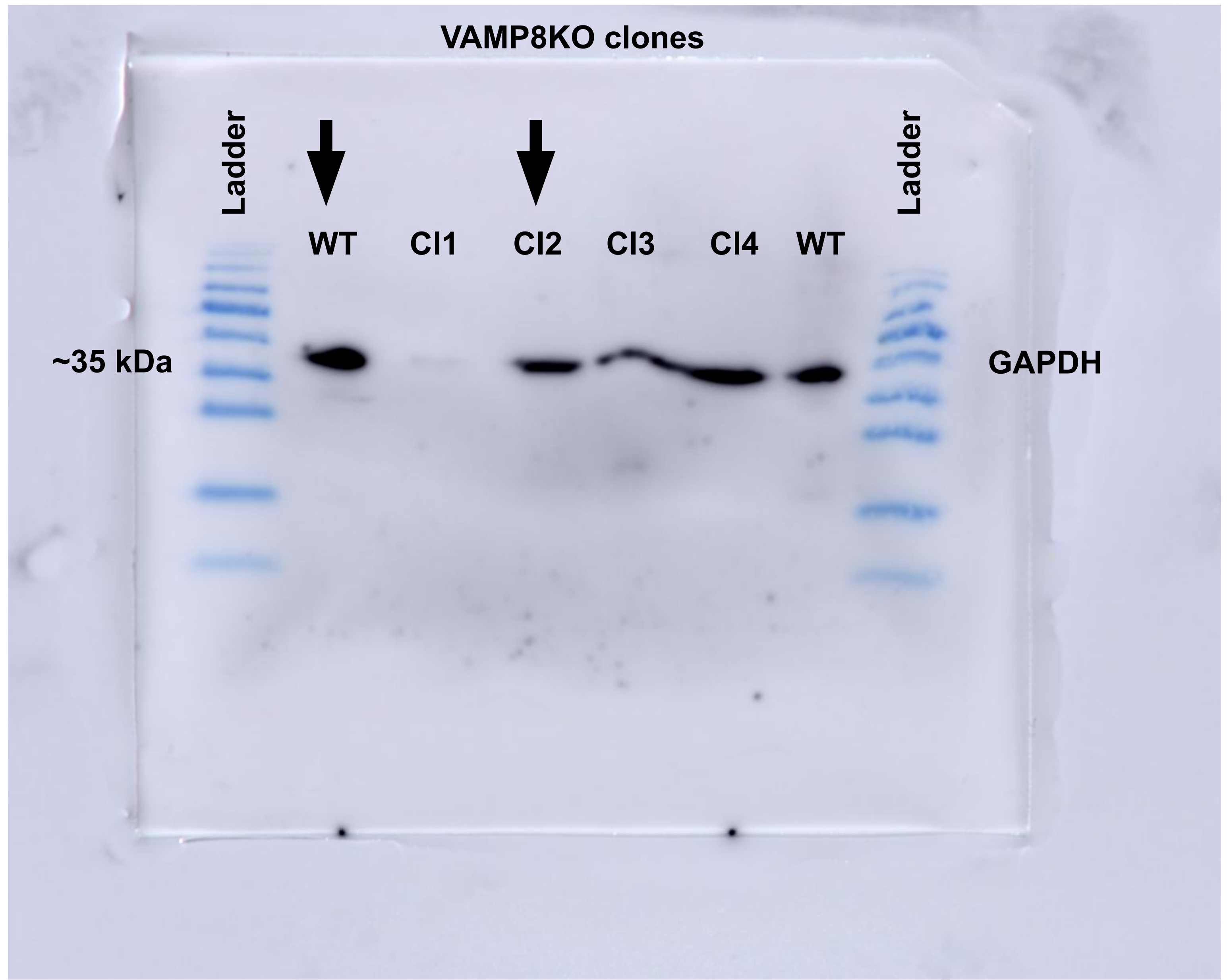

Used in the publication

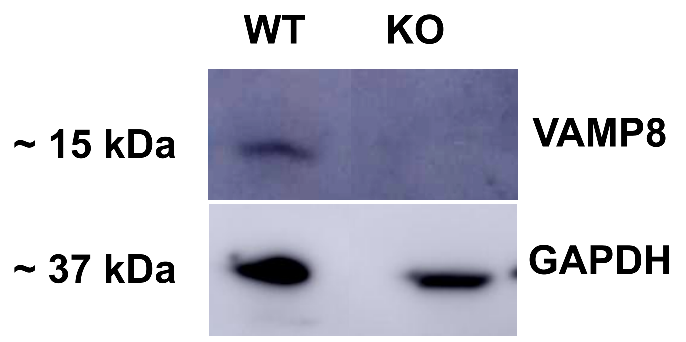

Stx7 KO clones WB

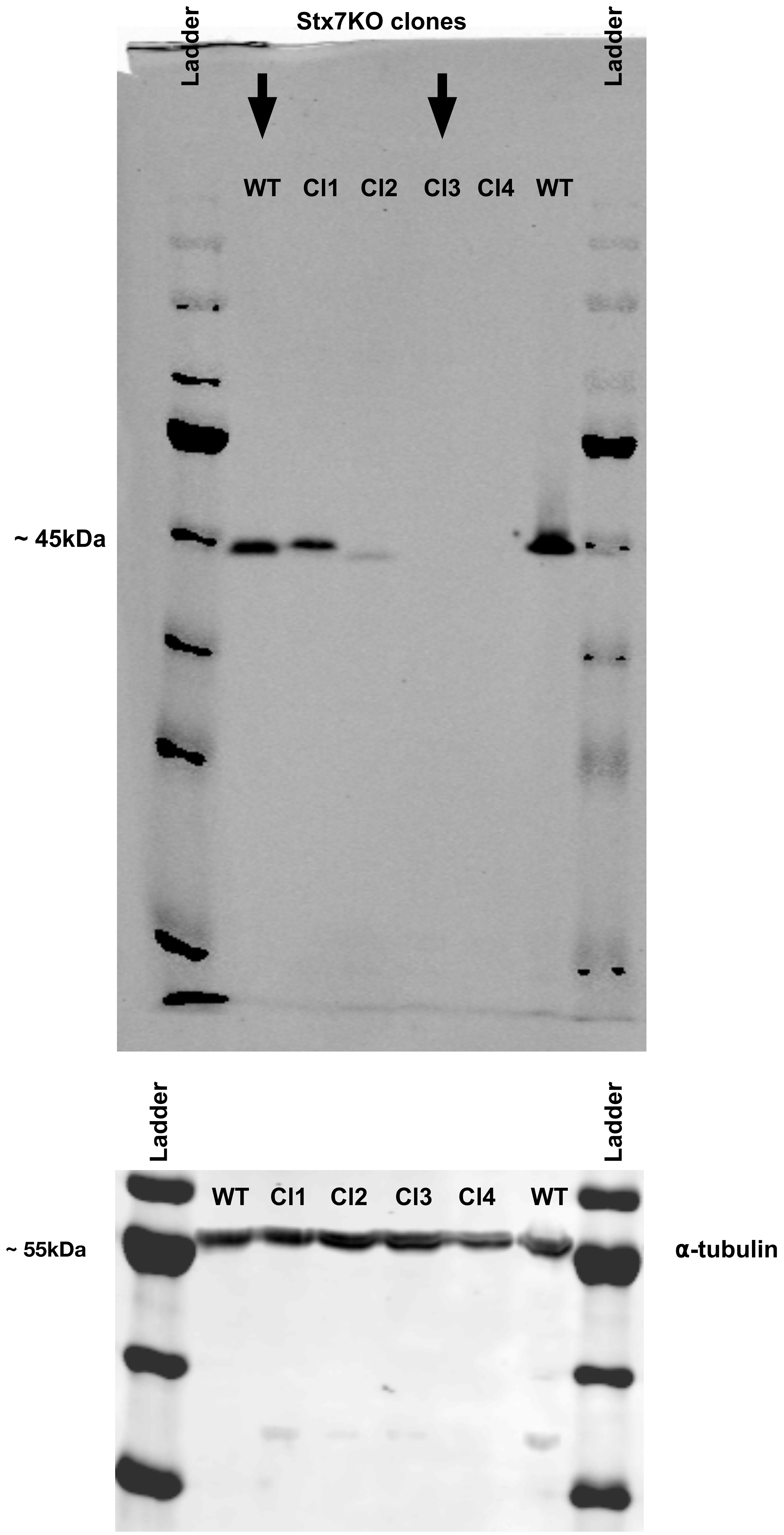

Used in the publication

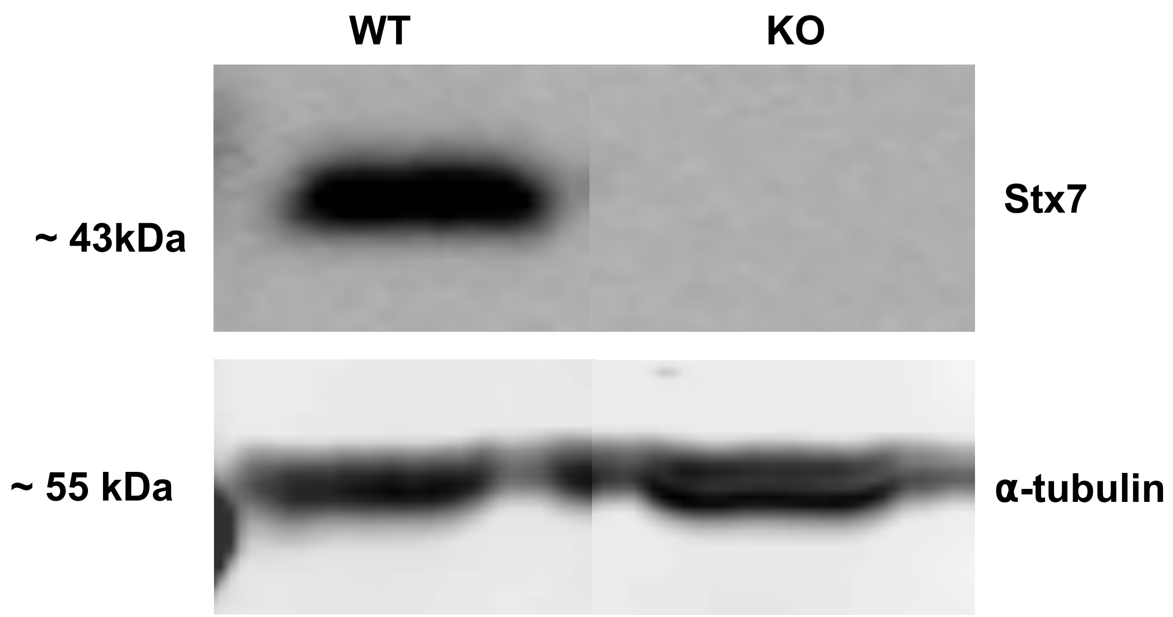

VAMP7-VAMP8 2KO WB

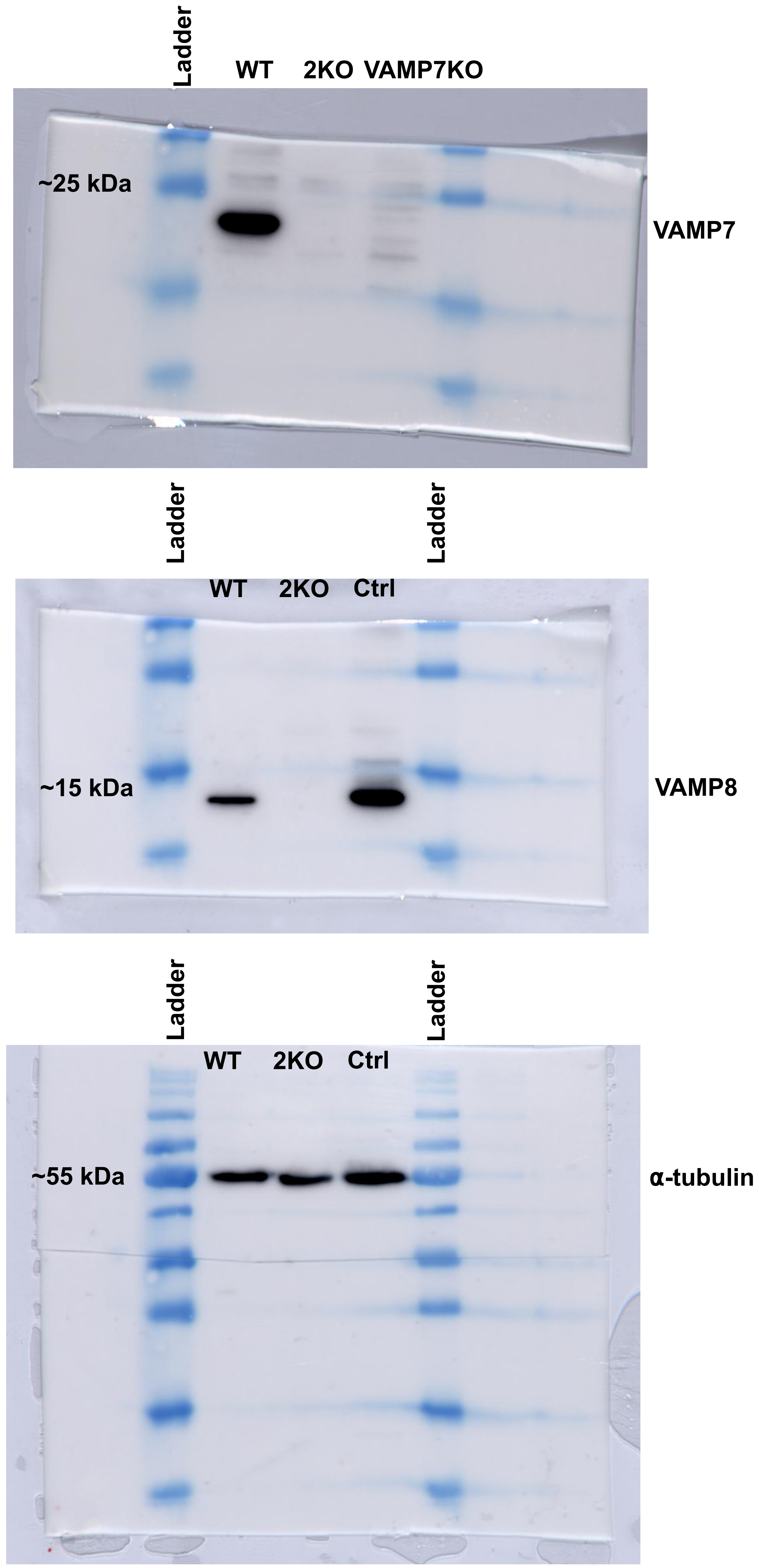

Used in the publication

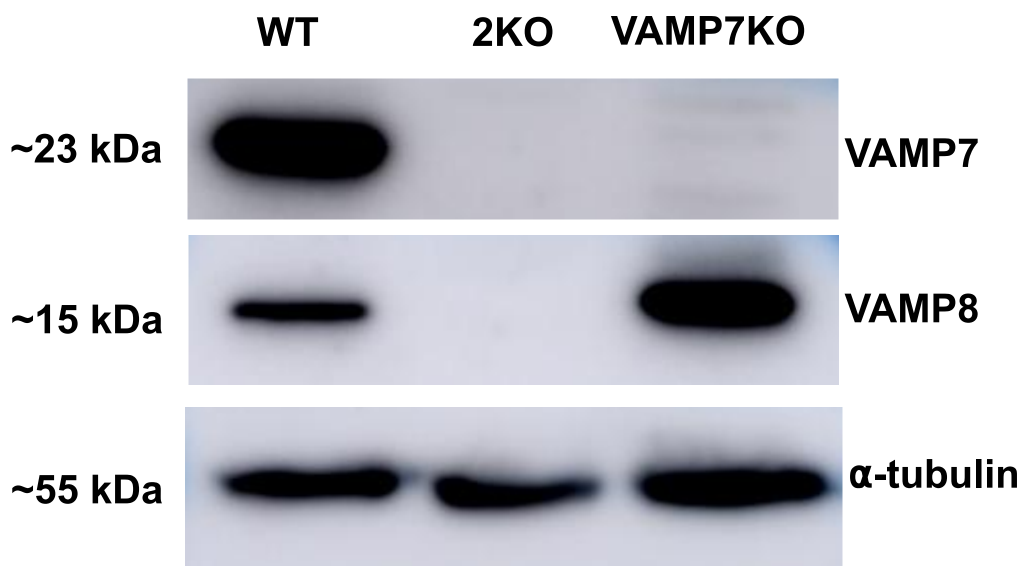

VAMP7-VAMP8-Vti1B 3KO clones WB

Clone used

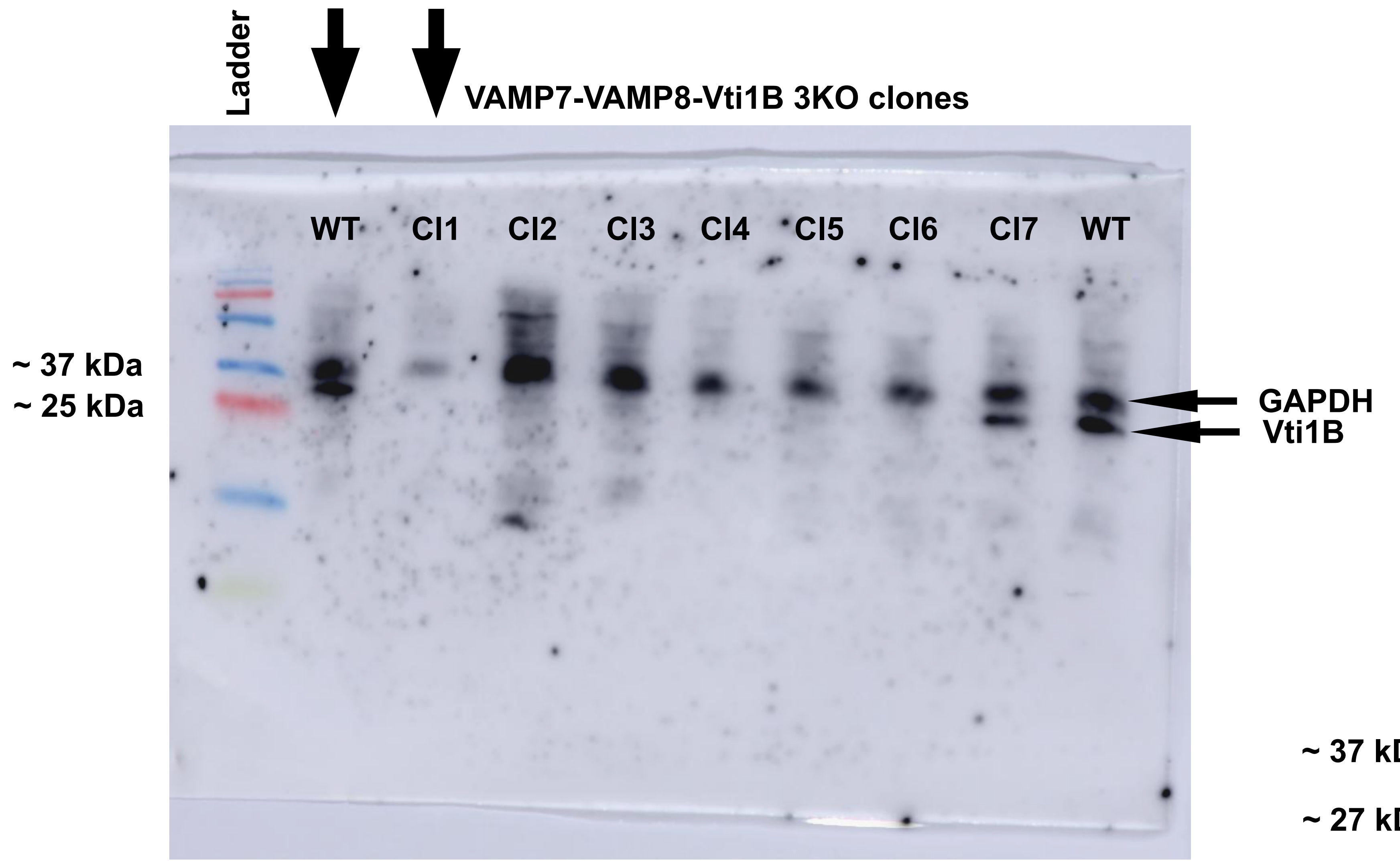

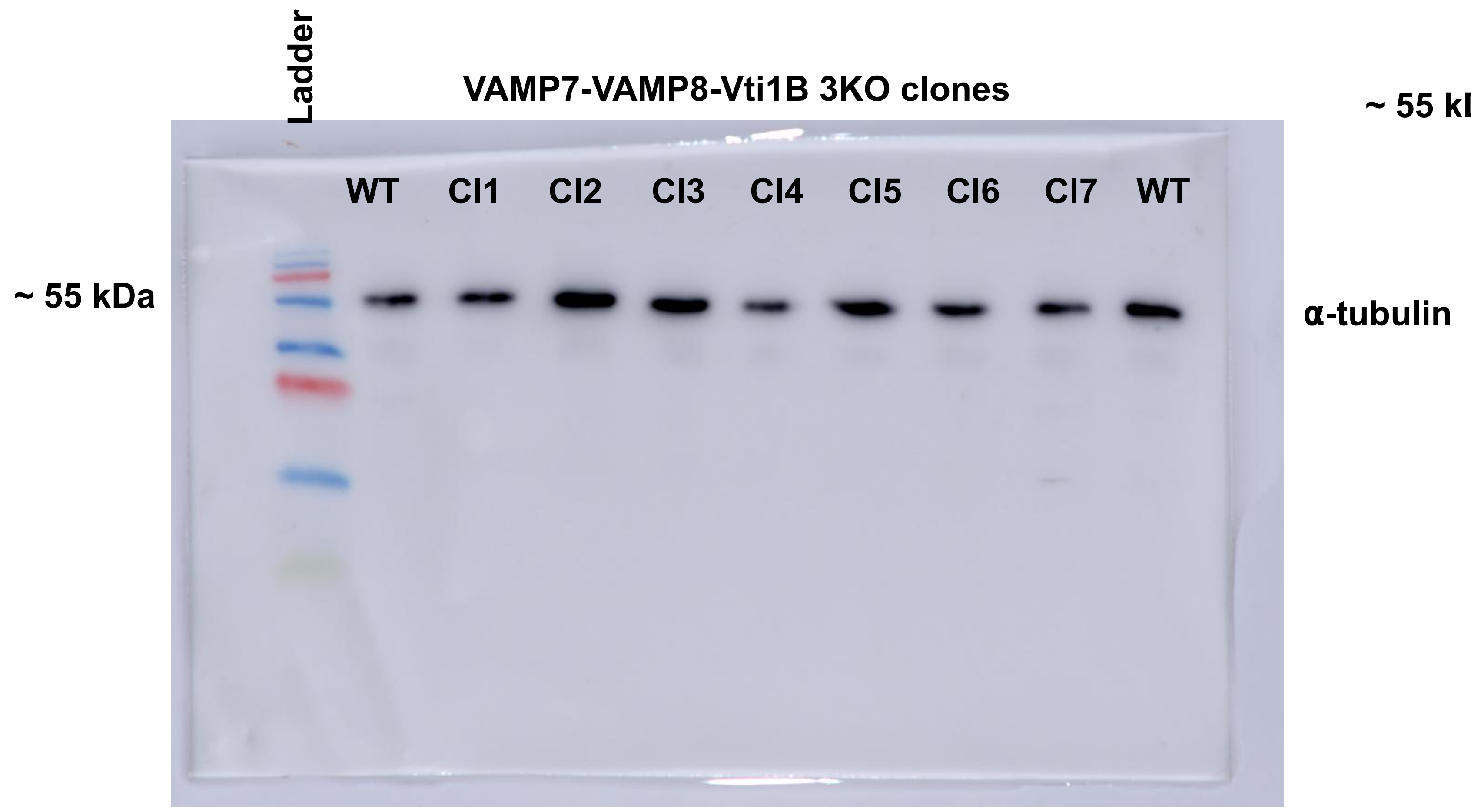

Used in the publication

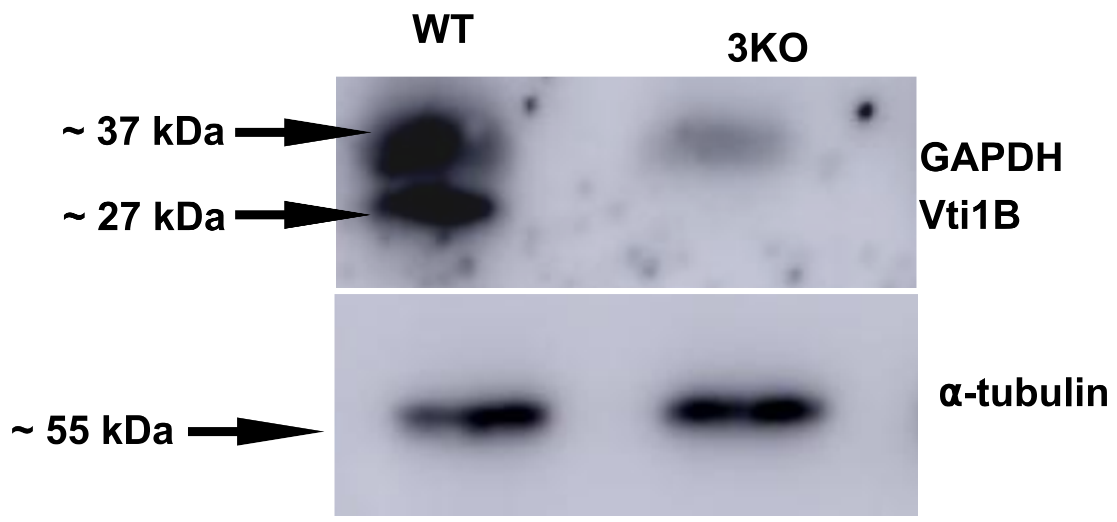

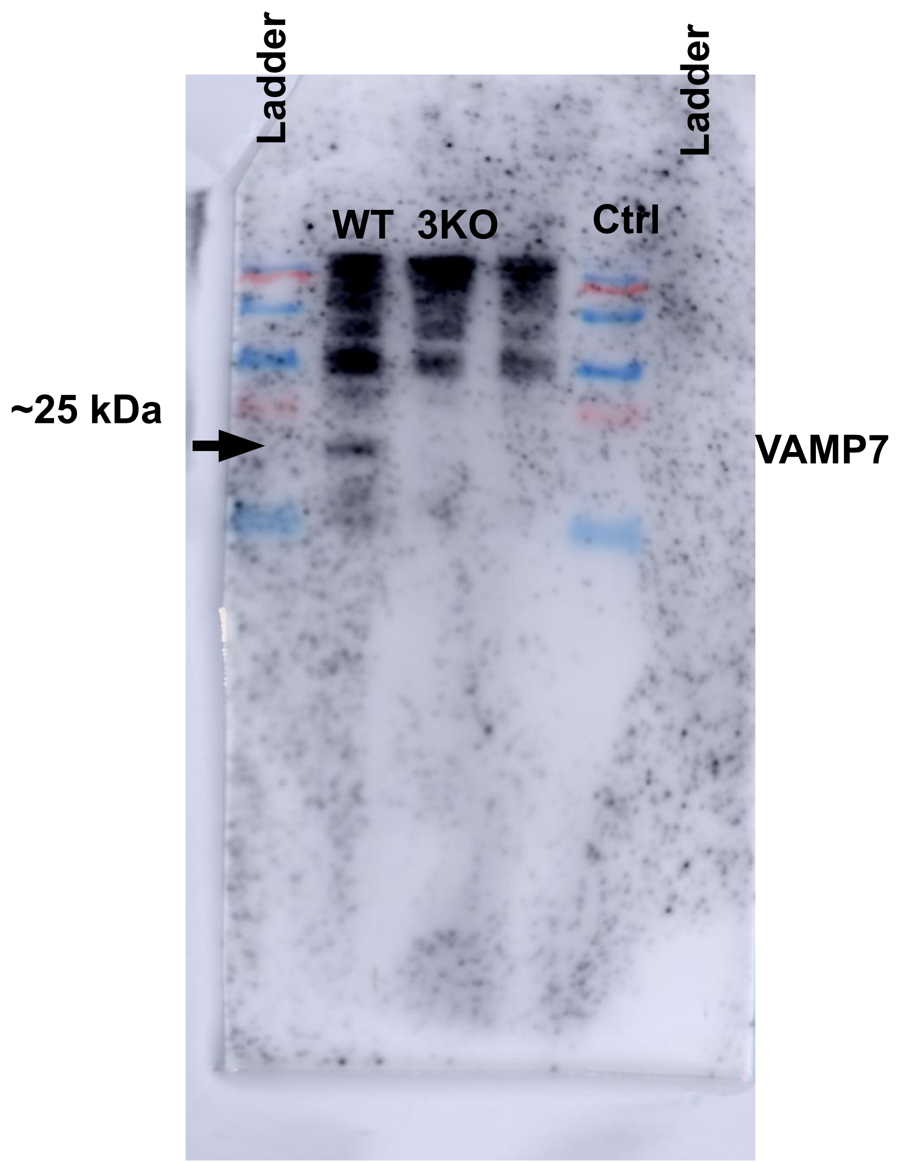

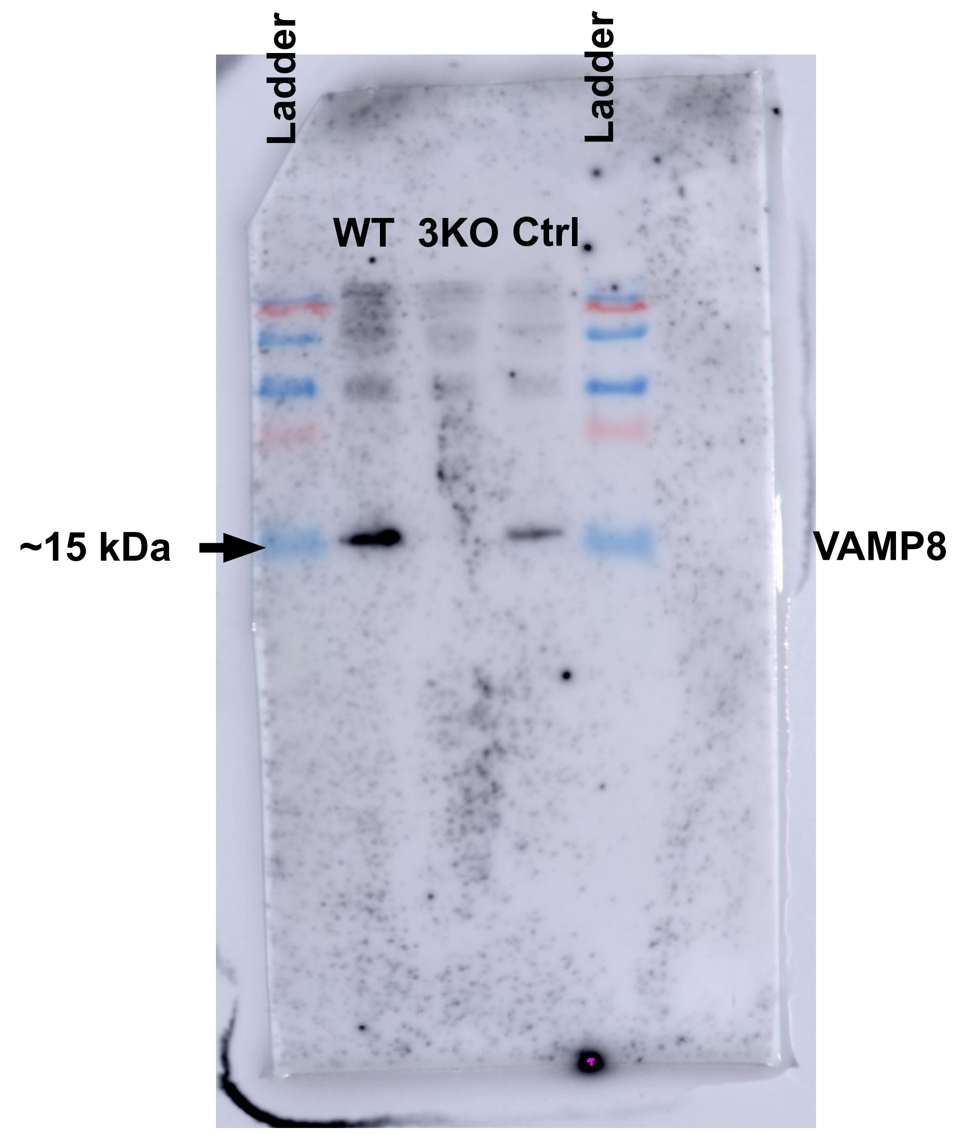

Ctrl = VAMP7KO

Used in the publication

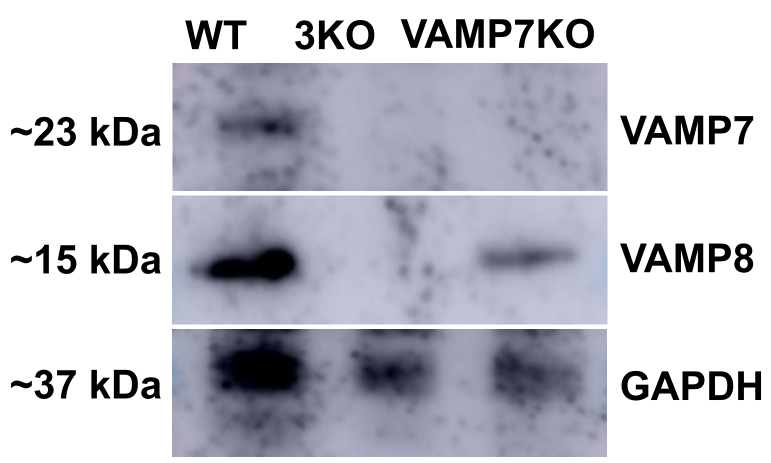

Supplement: Supplementary file 1 [file cells-15-00584-s001.zip › Supplementary Figure S3.pdf]
