# Supplementary figures and images for: Host SNARE Proteins Mediate Lysosome and PVM Fusion to Support Plasmodium Liver Infection
Source: Cells. 2026 Mar 25;15(7):584. doi: 10.3390/cells15070584 (PMC13072298; doi:10.3390/cells15070584)

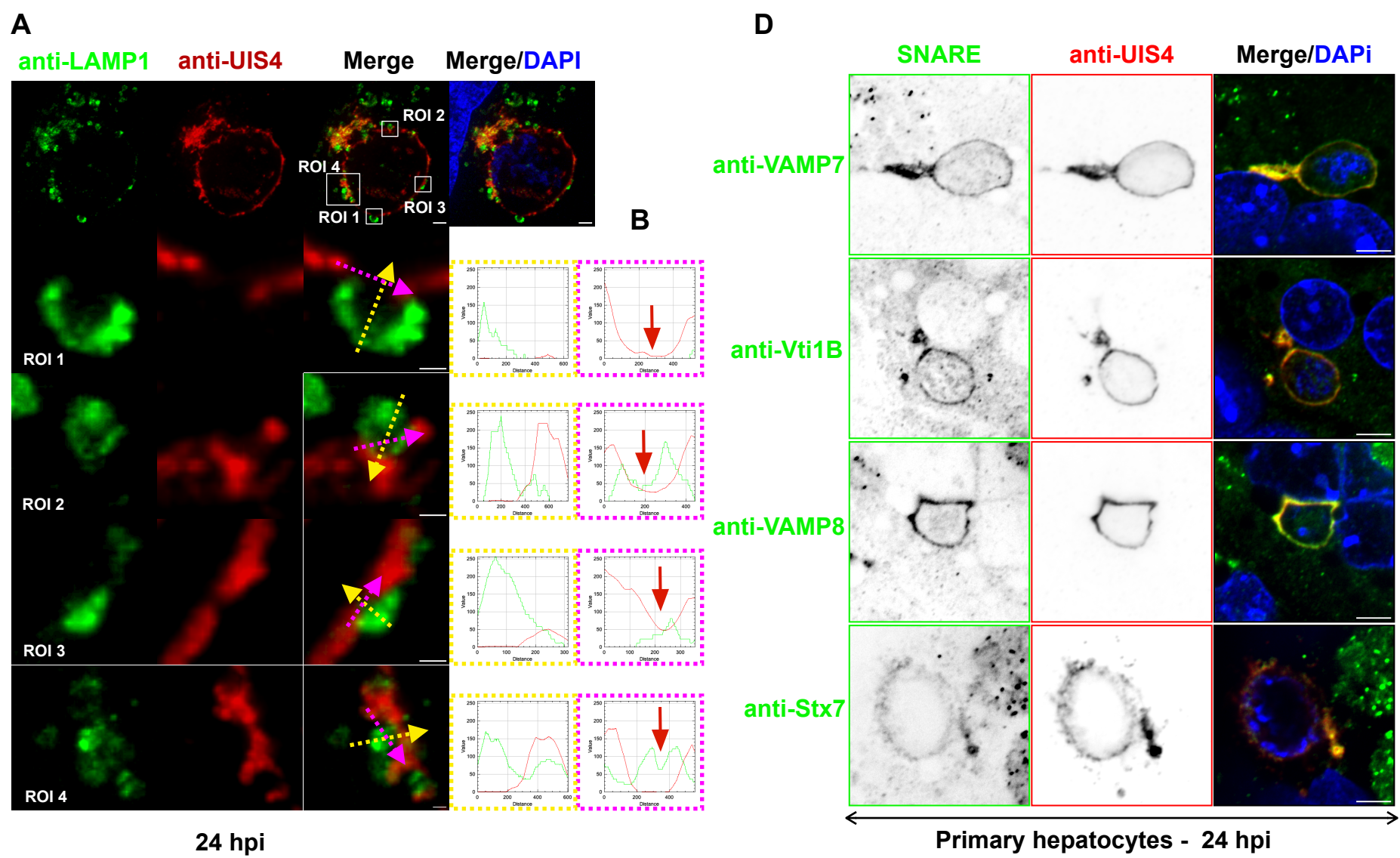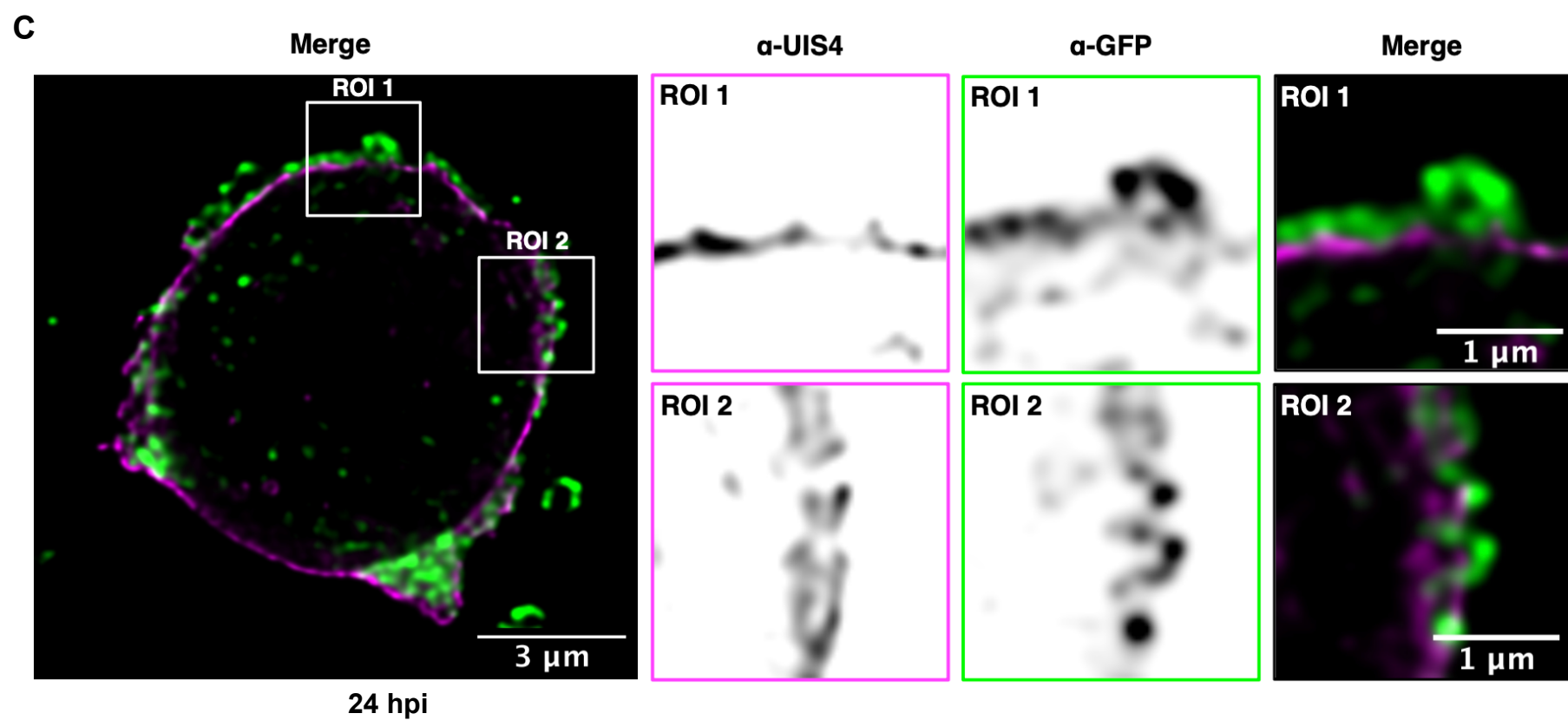

Supplement: Supplementary file 1 [file cells-15-00584-s001.zip › Supplementary Figure S1.pdf]

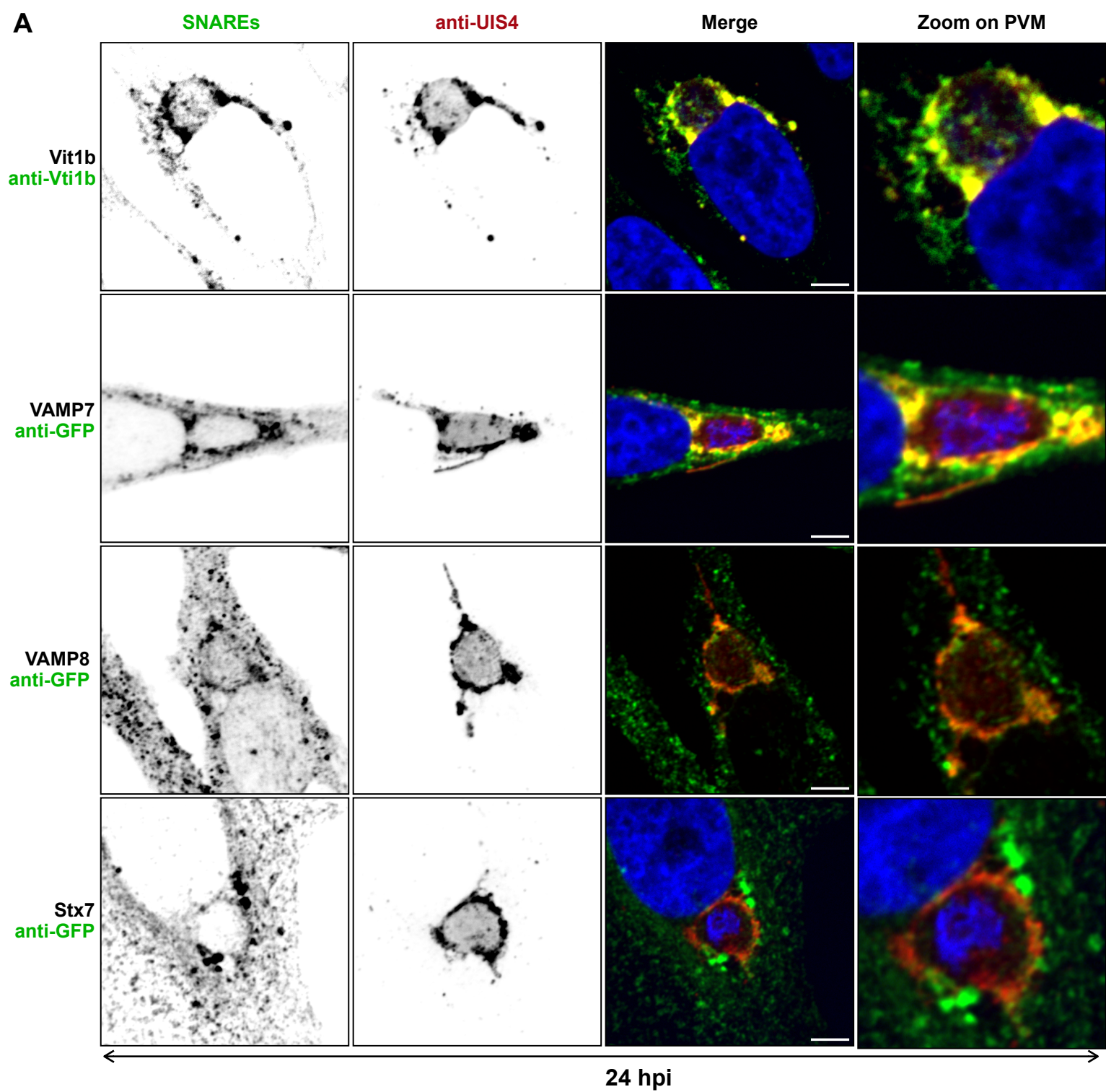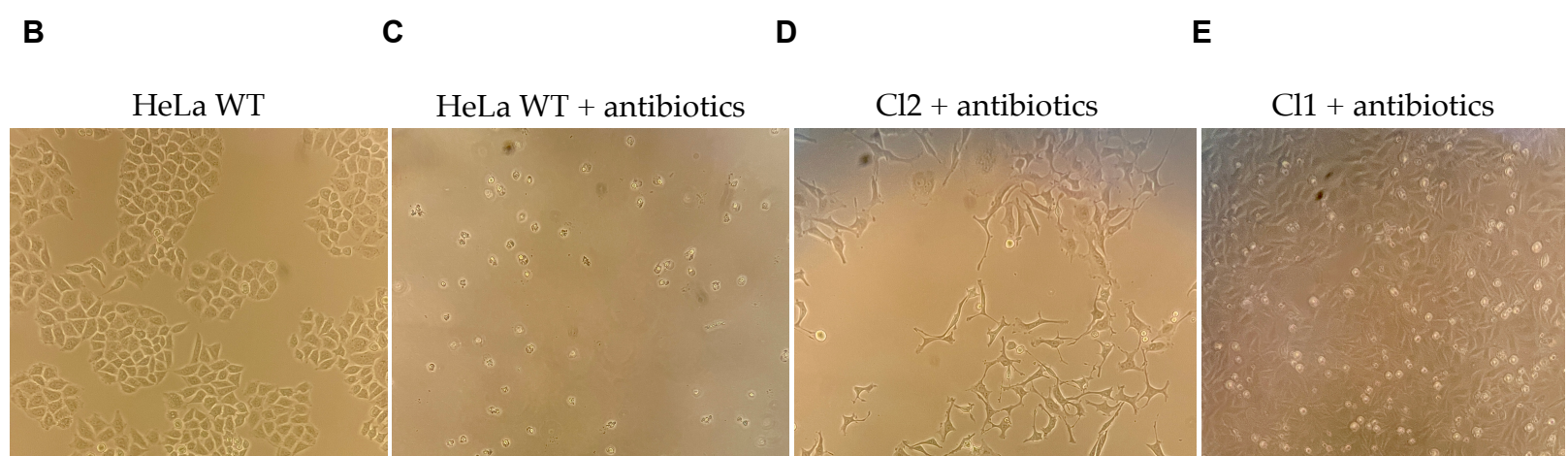

Antibiotics : Puromycin, Hygromycin and Zeocin

Supplement: Supplementary file 1 [file cells-15-00584-s001.zip › Supplementary Figure S2.pdf]
